# Monitoring flux in signalling pathways through measurements of 4EBP1-mediated eIF4F complex assembly

**DOI:** 10.1186/s12915-019-0658-0

**Published:** 2019-05-22

**Authors:** Yuri Frosi, Rachael Usher, Dawn Thean Gek Lian, David P. Lane, Christopher J. Brown

**Affiliations:** p53 Laboratory, A*STAR (Agency for Science, Technology and Research), 8A Biomedical Grove, #06-04/05, Neuros/Immunos, Singapore, 138648 Singapore

## Abstract

**Background:**

The most commonly occurring cancer mutations, including oncogenes such as MYC, Ras and PIK3C, are found in signal transductions pathways feeding into the translational machinery. A broad range of translation initiation factors are also commonly found to be either amplified or mis-regulated in tumours, including eIF4E (elongation initiation factor 4E). eIF4E is a subunit of the eIF4F protein initiation complex and required for its recruitment. Here we measure the formation of the eIF4F complex through interactions of eIF4E and eIF4G subunits, and the effect of oncogenic signalling pathways on complex formation.

**Results:**

We developed a protein fragment complementation (PCA) assay that can accurately measure the status of the eIF4E-eIF4G interaction in cells and quantify the signalling flux through the RAS/ERK and PI3K/AKT pathways regulating eIF4F assembly. Complex disruption induced by inhibition of either pathway was shown to be a function of the phosphorylation status of 4EBP1, a key mediator of eIF4F assembly that interacts directly with eIF4E, confirming 4EBP1’s ability to integrate multiple signals affecting cap-dependent translation. Maximal measured disruption of the eIF4F complex occurred under combined mTORC1 and mTORC2 inhibition, whilst combined inhibition of both RAS/ERK and PI3K/AKT pathways in parallel resulted in greater inhibition of eIF4F formation than individually. v-Myc-mediated resistance to dual mTORC/PI3K inhibition was also principally demonstrated to depend on the lack of competent 4EBP1 available in the cell to bind eIF4E.

**Conclusions:**

We show that 4EBP1 is a critical regulator of the mitogen responsive RAS/ERK and PI3K/AKT pathways and a key transducer of resistance mechanisms that affect small molecule inhibition of these pathways, principally by attenuating their effects on cap-dependent translation. These findings highlight the importance of highly efficacious direct inhibitors of eIF4E and eIF4F assembly, which could potentially target a wide spectrum of tumours containing differing mutations that effect these pathways and which confer chemo-resistance.

**Electronic supplementary material:**

The online version of this article (10.1186/s12915-019-0658-0) contains supplementary material, which is available to authorized users.

## Background

Mutational activation of mitogenic signalling is a common event in human cancer and is frequently found in genes that encode components of the PI3K/AKT/mTOR and the RAS/RAF/MEK/ERK pathways [1, 2]. Mutations in both pathways often co-exist. Activation of these pathways induces changes in transcription, metabolism, proliferative rate, protein synthesis and other processes that contribute to cellular transformation [1, 2]. Both these pathways converge to regulate the formation of the eukaryotic translation initiation complex 4F (eIF4F) that consists of three subunits eIF4E, eIF4A and eIF4G, which binds to the 7-methylguanylate cap (m^7^G) at the 5′ end of messenger RNA, thereby modulating the translation of a specific subset of cap-dependent sensitive mRNAs (c-myc, cyclin-D1) [3]. eIF4E interacts directly with the 5′ cap structure of mRNA, whilst the eIF4A helicase unwinds the secondary structure present in the 5′UTR of mRNAs and eIF4G, which interacts with both eIF4A and eIF4E, recruits eIF3 and interacts with PABP (polyadenylate-binding protein) [4]. The interaction of eIF4G with the multi-component initiation factor eIF3 enables the recruitment of the 43S pre-initiation complex and allows it to transverse the 5′UTR of the mRNA until it encounters the AUG start codon and halts. Here, formation of the 80S ribosomal complex occurs and translation is initiated [4, 5].

The activity and formation of the eIF4F complex is strictly regulated by the 4E-binding protein family (4EBP1, 4EBP2 and 4EBP3) [6–8]. The 4EBPs negatively regulate the assembly of eIF4F from its components by inhibiting the interaction of eIF4E with eIF4G. The 4EBPs share a common “canonical” primary binding motif with eIF4G (YXXXXLΦ, X = any amino acid and Φ = hydrophobic amino acid) to eIF4E, which allows them to competitively displace the eIF4G scaffold protein [9]. Structural studies have also established the presence of a non-canonical binding motif in the 4EBPs downstream of their primary interaction site with eIF4E. Although this secondary binding site shares little sequence similarity across orthogonal species, the lateral surface of eIF4E where it interacts is conserved. However, despite the non-canonical site not being required for the interaction of the 4EBPs with eIF4E, it does play a critical role in ensuring that the 4EBPs efficiently compete with eIF4G for binding to eIF4E and repress translation [10–14].

The displacement of eIF4G impairs the assembly of the eIF4F complex on the 5′ cap structure and hinders cap-dependent translation. The actions of 4EBPs are controlled by their phosphorylation status, which is under the specific control of mTOR [15, 16]. AKT performs a significant role in regulating mTOR activity [17, 18]. eIF4E is also regulated via the MAPK (mitogen activated protein kinase) and RAS/ERK pathways through direct phosphorylation by the MAPK-interacting kinases (Mnk1 and Mnk2) of Ser209 [19]. Direct phosphorylation of eIF4E also plays an important role in cancer development, where mouse studies have demonstrated that mutant eIF4E^S209A^ mice, which are incapable of being phosphorylated by the MNK kinases (1/2), are resistant to polyoma middle-T driven mammary tumours [20]. However, the precise effect of this post translational modification on the eIF4F complex is unclear [21].

Several strategies exist for targeting the eIF4F complex, which lies at a critical nodal point for the regulation of cap-dependent translation of the major cancer signalling pathways [22]. One approach is to target eIF4F formation with inhibitors of the PI3K/AKT/mTORC pathway [23]. These inhibitors induce de-phosphorylation of the 4EBPs and cause the dissociation of eIF4E from eIF4G [22]. Another approach is to inhibit phosphorylation of eIF4E itself through the use of MNK1/2 kinase antagonists in the RAS/ERK signalling pathway [22]. Emerging resistance mechanisms to these therapeutic interventions are increasingly being reported. Cells that become resistant to the dual mTORC/PI3K inhibitor BEZ235 were shown to contain amplified c-Myc and eIF4E genes [24]. Additionally, cells that acquired resistance to the mTORC1 and mTORC2 active site inhibitor AZD8055 also contained amplified eIF4E [25]. Additionally, resistance to mTORC inhibition and preservation of the eIF4F complex has also been shown to include loss of expression of individual members of the 4EBP protein family [26–28]. These findings underline the importance of eIF4F complex disruption in mediating the actions of these therapies. Other strategies that have been pursued to interfere with the activity of the eIF4F complex are the development of ASOs (antisense oligonucleotides) to reduce eIF4E expression [22], the discovery of chemical inhibitors of the eIF4A helicase [22] and exploration of cap analogues to prevent recruitment to capped mRNA [22, 29, 30].

The interface between eIF4G and eIF4E can also be directly targeted with small molecules and peptides [22, 31, 32]. Protein-protein interfaces (such as eIF4E:4G), which are devoid of hydrophobic clefts and are relatively large and planar, are considered intractable to small molecule (< 500 MW) development. Currently, there is a great deal of interest in developing novel modalities (e.g. peptide and non-peptidic macrocycles) that can form much larger interaction interfaces with their target molecules than small molecules and can therefore disrupt protein-protein interactions more efficiently [33]. However, many of these molecules are not inherently cell permeable and require significant chemical optimisation or the development of new cell delivery methods for their utilisation. Several systems have been developed to measure eIF4E/eIF4G complex assembly in response to drug treatment, such as proximity ligation assay (PLA) [34] and time-resolved – fluorescence resonance excitation transfer (TR-FRET) [35]. However, these assays are limited as they cannot be performed on live cells. Therefore, there is a need for the development of new assays to quantitatively measure the ability of these compounds to permeate into live cells and to directly engage the target of interest. Additionally, it is critical to ensure that this occurs in the absence of non-specific effects, e.g. cell membrane perturbation [36].

In this work, a NanoLuc (PROMEGA, Ltd., USA)-based protein fragment complementation assay (PCA) [37] has been developed and validated that allows the integrity of the eIF4F complex through the eIF4E:eIF4G interaction to be monitored in live cells. More importantly, the eIF4E:eIF4G PCA also enables the direct assessment of novel inhibitors and resistance mechanisms on the assembly of the eIF4F complex and also allows the delineation of their precise effect on cap-dependent translation.

## Results

### Detecting the eIF4E-eIF4G interaction with a NanoLuc-based protein complementation assay

**Fig. 1:**
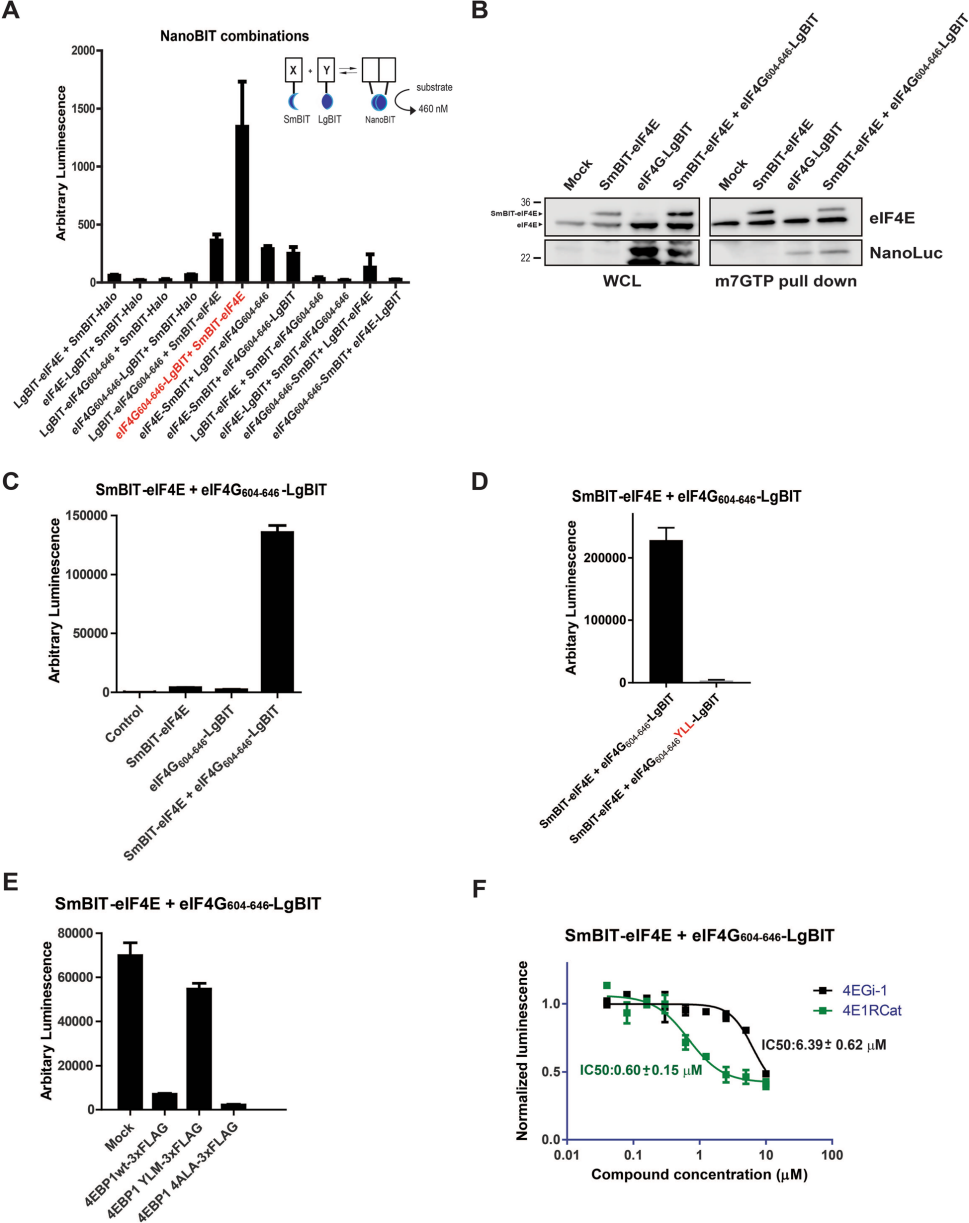
**a** Inset shows how the interaction of proteins A and B enables their SmBiT and LgBiT fusions (Promega, USA) to be brought into close proximity to each other, allowing complex formation to occur and reconstitution of the active NanoBit (Promega, USA) luciferase. Graph shows the reconstituted luminescence activity of the various combinations of potential interacting N- and C-terminal-fused SmBiT and LgBiT constructs linked either to full-length eIF4E or the eIF4G^604–646^ fragment, respectively, co-transfected into HEK293 cells. Individual N- and C-terminal LgBiT-linked eIF4E and eIF4G constructs co-transfected with SmBiT-HALO served as negative controls. **b** Western blot analysis of HEK293 cells, either co-transfected with SmBiT-eIF4E and eIF4G^604–646^-LgBiT or with each construct alone in combination with empty vector DNA, in whole cell lysate (Left, WCL) and with m^7^GTP pulldown analysis (right). Mock control consisted of co-transfection of empty SmBiT and LgBiT vectors. eIF4E and SmBIT-eIF4E (highlighted with black arrows) or eIF4G^604–646^-LgBiT were detected by using anti-eIF4E and anti-NanoLuc antibodies. **c** SmBiT-eIF4E and eIF4G^604–646^-LgBiT constructs were either co-transfected together or separately co-transfected with corresponding empty vector DNA. Mock control consisted of co-transfection with both empty SmBiT and LgBiT vectors into HEK293 cells. **d** SmBiT-eIF4E was co-transfected with either eIF4G^604–646^-LgBiT or eIF4G^604–646(YLL)^-LgBiT constructs. **e** SmBiT-eIF4E and eIF4G^604–646^-LgBiT constructs co-transfected into HEK293 cells with either empty vector (mock) or vectors containing 4EBP1 wild-type or 4EBP1 mutants (as indicated). **f** 4EGi1 and 4E1RCat were titrated onto HEK293 cell co-transfected with SmBIT-eIF4E or eIF4G^604–646^-LgBiT. Luciferase activity was measured after 4 h. Luminescence signals were normalised with those obtained from DMSO vehicle-treated cells. Compound titrations were also normalised with respect to the counter screen titrations of the compounds against full-length NanoLuc (Additional file 1: Figure S1). All values represent mean ± SD (*n* = 3). The molecular mass of the protein marker is indicated in *K*_*d*_

We sought to determine the levels of eIF4F assembly in live cells by applying the novel NanoLuc-based protein complementation system (NanoBit, PCA) [37] to measure the eIF4E-eIF4G interaction (Fig. 1a, inset). The NanoLuc complementation protein system consists of two components termed LgBiT (18-kDa protein fragment) and SmBiT (11-amino-acid peptide fragment), which have been optimised for minimal self-association and stability. When LgBiT and SmBiT are optimally fused to two interacting proteins, they will be brought into close proximity to each other by the resulting interaction, whereupon they will form the active luciferase and generate a bright luminescent signal. Full-length eIF4E and the eIF4E interaction domain from eIF4G (604–646) were selected for the construction of the PCA system to measure eIF4F complex formation. All eight possible combinations of eIF4E and eIF4G (604–646) fused to either the N- and C-termini of SmBiT and LgBiT were cloned into mammalian expression vectors. Each of the resulting possible interaction pairs was then co-transfected into HEK293 cells to identify the pair that enabled the LgBiT and SmBiT fusions to reconstitute the active luciferase (Fig. 1a). In order to determine that any measured luciferase signal arising from complementation of the SmBiT and LgBiT fragments was driven by the specific interaction of eIF4E with eIF4G (604–646), negative control experiments were performed by co-transfections of the relevant eIF4G/eIF4E-LgBiT construct with a negative control vector containing SmBiT only fused to the Halotag protein. Negligible background luciferase signal was measured for all of the interaction pairs tested, confirming that the NanoBit reporter fragments were not spontaneously assembling under the experimental conditions (Fig. 1a). The interaction pair consisting of the eIF4G-LgBiT and SmBiT-eIF4E fusion constructs was selected for further validation as it gave the highest luminescence signal in comparison to background luciferase activity measured in the negative control experiment.

The cellular co-expressions levels of the eIF4G-LgBiT and SmBiT-eIF4E interaction pair (now termed NanoBit eIF4E:eIF4G^604–646^ system) in HEK293 cells were also probed, revealing that SmBiT-eIF4E protein levels were reproducibly less abundant in comparison to endogenous eIF4E (Fig. 1b). Furthermore, luciferase activity of eIF4G-LgBiT and SmBiT-eIF4E were measured separately from each other confirming that only when co-expressed with each other could a luminescence signal be observed (Fig. 1c) and that the individual components of the eIF4E:eIF4G^604–646^ system were inactive and incapable of generating luciferase activity. Specific assembly of SmBiT-eIF4E with eIF4G-LgBiT to form the eIF4E:eIF4G^604–646^ complex was also detected via immunoprecipitation with m^7^GTP beads, confirming that SmBiT-eIF4E retained the ability to interact with the cap analogue found at the 5′UTR of eukaryotic mRNA (Fig. 1b). The three residues (Y624, L629 and L630) critical for eIF4G binding to eIF4E were all mutated to alanine in the eIF4G-LgBit^YLL^ construct; this resulted in a dramatic reduction in the luciferase signal when the eIF4G-LgBit^YLL^ was co-transfected with SmBIT-eIF4E (Fig. 1d). Also no eIF4G-LgBit^YLL^ protein could be detected in complex with eIF4E after m^7^GTP immunoprecipitation (Additional file 1: Figure S1A). These results confirm that the luciferase signal is the product of specific complementation between the eIF4G-LgBit and SmBIT-eIF4E expressed in cells.

eIF4E and eIF4G complex formation occurs through the eIF4E interaction recognition motif found in eIF4G, which is competitively regulated by the eIF4E-binding proteins (4EBPs). The 4EBPs all share the same eIF4E interaction motif with eIF4G. To confirm that the NanoBit eIF4E:eIF4G^604–646^ system was completely functional, co-expression experiments were performed in mammalian cells with 4EBP1 constructs, which were either competent or unable to interact with eIF4E (Additional file 1: Figure S1B and S1C), to demonstrate that the NanoBit eIF4E:eIF4G^604–646^ signal could be decreased by specific disruption of the eIF4E:4E complex. Two constructs, 4EBP1^WT^ and 4EBP1^4ALA^, were utilised to target the NanoBit eIF4E:eIF4G^604–646^ complex, both of which significantly reduced the measured luminescence signal, with 4EBP1^4ALA^ exhibiting the largest amount of inhibition (Fig. 1e). The 4EBP1^ALA^ in contrast to the wild-type construct 4EBP1^WT^ has all four phosphorylation sites (Additional file 1: Figure S1D), which are responsible for negatively regulating binding of 4EBP1 to eIF4E, mutated to alanine allowing the NanoBit signal to be more efficiently disrupted. In addition, no significant reduction in luminescence was observed with co-expression of the NanoBit eIF4E:eIF4G^604–646^ complex with the negative construct 4EBP1^YLM^, where the eIF4E interaction motif in 4EBP1 has been abrogated and its binding to eIF4E abolished (Additional file 1: Figure S1C), confirming the specific disruption observed for 4EBP1^ALA^ and 4EBP1^WT^ (Fig. 1e). These results also verify that the observed NanoBit complementation is driven by the specific molecular interaction between SmBiT-eIF4E and eIF4G-LgBiT.

Several small molecules also have been reported to interfere with eIF4F signalling and cell proliferation by disrupting the eIF4E-eIF4G interface directly (4ER1Cat) or via an indirect allosteric mechanism (4EGi-1). To further demonstrate the versatility of this intracellular PPI assay, both these molecules were titrated onto the NanoBit eIF4E:eIF4G^604–646^ system where a corresponding dose-dependent inhibition was observed in the NanoBit signal (Additional file 1: Figure S1E). To ensure that these compounds were specifically disrupting the eIF4E:4G interaction, 4ER1Cat and 4EGi-1 were also titrated onto cells transfected with the full-length luciferase, which is re-constituted by the NanoBit protein complementation system. Non-specific inhibition of the luciferase signal was observed for both compounds (Additional file 1: Figure S1F). This decrease in luminescence was due to absorbance of the luciferase signal by the compounds themselves. Compound titrations in the NanoBit system were therefore normalised with respect to the full-length luciferase counter-screen and the following IC_50_s were respectively determined, 0.60 ± 0.15 μM (4E1RCat) and 6.39 ± 0.62 μM (4EGi-1) (Fig. 1f). 4ER1Cat and 4EGi-1 were also tested for their effects on cell viability, where only 4ER1Cat was observed to have an effect over the 4-h treatment period (Additional file 1: Figure S1G). These results correlated with the rank order of the in vitro *K*_*d*_ values published for both these compounds, 3.2 μM (4E1RCat, [35]) and 25 μM (4EGi-1, [38]). Interestingly both compounds were much more potent in the NanoBit cell-based assay, and neither achieved complete disruption of the signal that was seen with the eIF4G^(Y624A, L629A, L630A)^-LgBit binding control.

### Measurement of intracellular eIF4E-eIF4G complex disruption by release of endogenous 4EBP1

In mammalian cells, eIF4F complex formation is principally regulated by the availability of un-phosphorylated 4EBP1, which is under the direct control of mTORC1. Hyper-activation of mTORC1 results in over-activation of the eIF4F complex due to hyper-phosphorylation of its negative regulator 4EBP1. mTORC1 is a target of multiple signalling pathways involved in cancer development, whose components as well as mTORC1 itself are also key targets for therapeutic development, e.g. ERK, AKT, PI3KC. Therefore, it is a key requirement for the use and applicability of the NanoBit eIF4E:eIF4G^604–646^ system to demonstrate that is it capable of detecting endogenous 4EBP1-mediated inhibition of the eIF4F complex resulting from mTORC1 inhibition. Two well-known classes of inhibitors exist for mTORC1, which are the rapalogs, e.g. Rapamycin and Everolimus [39], and the ATP competitive-based inhibitors, e.g. Torin [40] and PP242 [41]. The rapalogs are allosteric inhibitors that interact with the protein FKBP12 and interact together to specifically bind mTORC1, but not mTORC2, at a site adjacent to the kinase active site. [19]. On the other hand, compounds like Torin and PP242 have been designed to specifically inhibit the catalytic activity of mTOR itself, allowing this class of compounds to efficiently inhibit the phosphorylation events catalysed by mTORC1 and mTORC2.

**Fig. 2:**
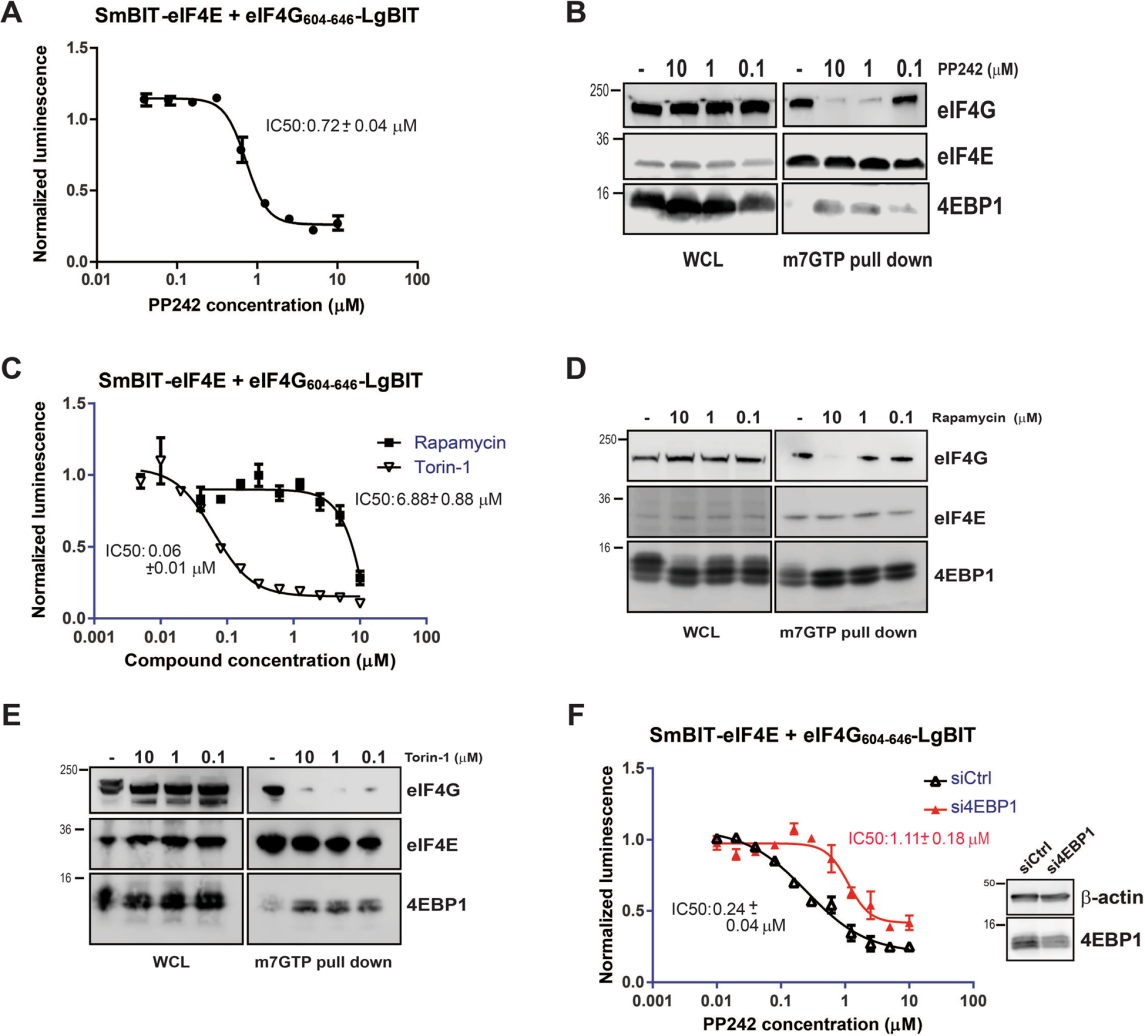
**a** Titration of the dual MTORC1/2 active site inhibitor PP242 onto HEK293 cells co-transfected with the NanoBit eIF4E:eIF4G^604–646^ system. **b** Western blot analysis of endogenous level of eIF4E, eIF4G and 4EBP1 in non-transfected 293FT extracts and associated m7GTP pulldowns of eIF4E containing complexes with differing treatment concentrations of PP242. **c** Titrations of the mTORC1 allosteric inhibitor Rapamycin and the potent mTORC1/2 active site inhibitor Torin onto HEK293 cells co-transfected with the NanoBit eIF4E:eIF4G^604–646^ system. Western blot analysis of endogenous level of eIF4E, eIF4G and 4EBP1 in non-transfected 293FT extracts and associated m7GTP pulldowns of eIF4E containing complexes with differing concentrations of **d** Rapamycin or **e** Torin**. f** HEK293 cells were co-transfected with the NanoBit eIF4E:eIF4G^604–646^ system together with either siRNA Ctrl (siCtrl) or si4EBP1 and PP242 titration was performed again as in **a**. The inset shows the level of 4EBP1 siRNA-mediated knockdown by western blot using anti-4EBP1 antibody. Beta-actin was visualised as loading control. IC_50_ values were determined for the titrations described in sections **a**, **c** and **f** using four parameter curve fits. Non-linear regression analysis was performed in GraphPad (Prism). All values represent mean ± SD (*n* = 3). The molecular mass of the protein marker is indicated in kilodaltons

PP242, Rapamycin and Torin were all used to verify the sensitivity of the NanoBit eIF4E:eIF4G^604–646^ system for these separate classes of mTOR inhibitors. The compounds were titrated onto NanoBit eIF4E:eIF4G^604–646^ co-transfected HEK293 cells, where their IC_50_s were determined to be 0.72 ± 0.04 μM (PP242), 6.88 ± 0.88 μM (Rapamycin) and 0.06 ± 0.01 μM (Torin), respectively (Fig. 2a, c). The dissociation of the NanoBit complex by these compound treatments also conclusively demonstrated that the complementation of the SmBiT and LgBiT components to form the luciferase does not result in the formation of a stable refolded reporter protein that cannot be disassembled after expression. m^7^GTP bead pulldowns of the eIF4F complex from un-transfected cells were performed at various concentration points corresponding to the beginning, midpoint and endpoint of the different NanoBit measured titration curves for each compound, which confirmed that the signal being measured by the NanoBit eIF4E:eIF4G^604–646^ system correlated to the disruption of the cellular eIF4F complex by dephosphorylated 4EBP1 (Fig. 2b, d and e). Further to this, specific siRNA-mediated knockdown of 4EBP1 protein levels attenuated the potency of PP242 fivefold in the NanoBit system, confirming the critical role of 4EBP1 in disruption of the eIF4E-eIF4G (and eIF4E:eIF4G^604–646^) complex via specific inhibition of the mTOR pathway (Fig. 2 f). Counter-screen titrations were also performed in HEK293 cells with the full-length luciferase reconstituted by the NanoBit eIF4E:eIF4G^604–646^ system, which demonstrated that none of the compounds tested inhibited the luciferase’s activity and additionally confirmed that the signal decrease resulted from specific disruption of the eIF4E:4G interaction (Additional file 2: Figure S2A and S2B). Furthermore, cell viability measurements of intracellular ATP levels showed that neither Rapamycin, Torin nor PP242 affected the cells adversely verifying the specificity of their effect in the NanoBit eIF4E:eIF4G^604–646^ system and that the decrease in luminescence is not due to cell death (Additional file 2: Figure S2C).

**Fig. 3:**
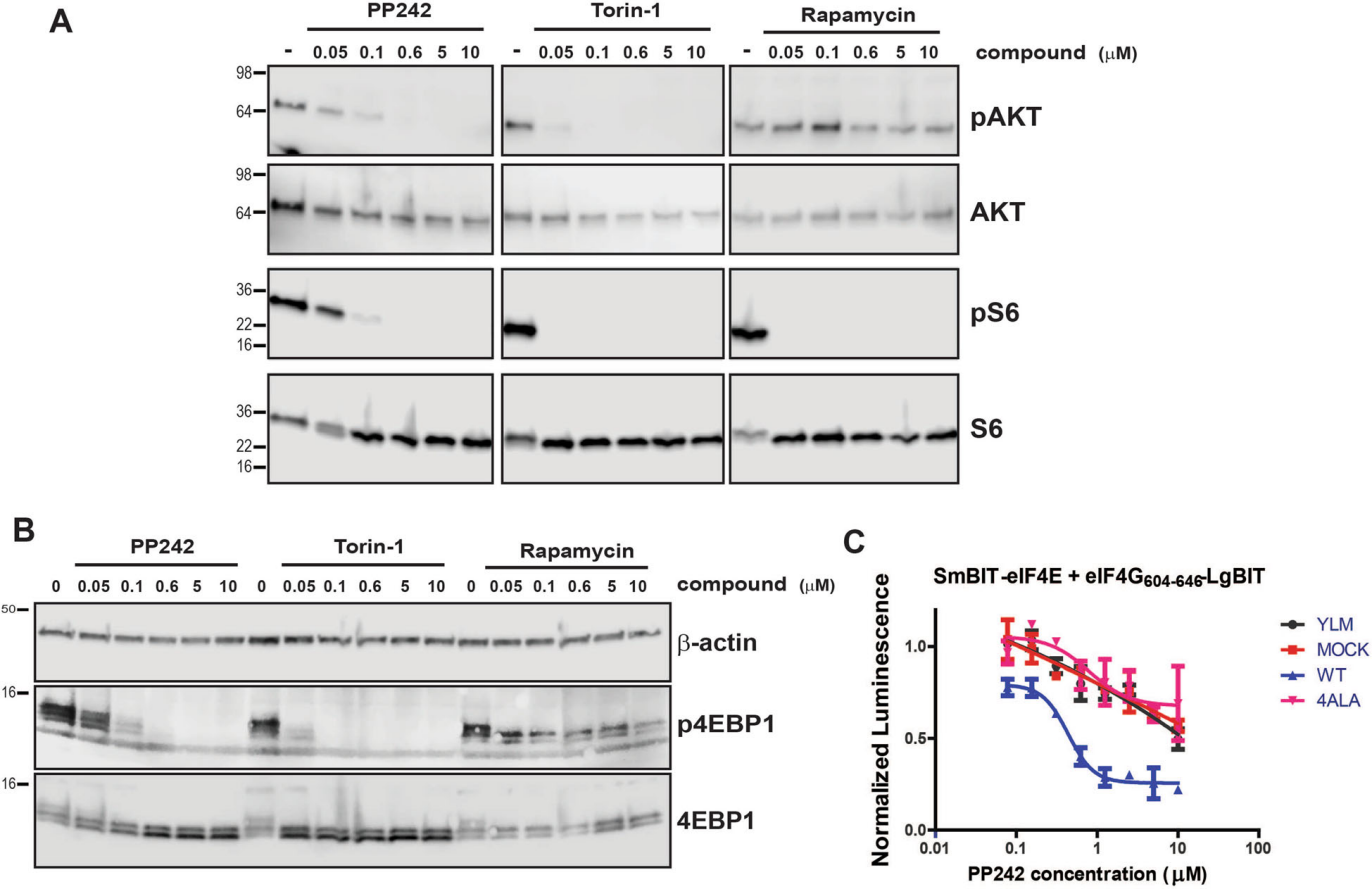
**a** Western blot analysis of endogenous phosphorylation status of AKT and S6 kinases in non-transfected HEK293 cells treated with either PP242, Torin, Rapamycin or the MNK1/2 inhibitor CGP57380. **b** Western blot analysis of endogenous 4EBP1 phosphorylation status in non-transfected HEK293 cells treated with either the dual mTORC1/2 active site inhibitors PP242 and Torin, or the allosteric inhibitor of mTORC1 Rapamycin **c** PANC1 cells, which contain low levels of endogenous 4EBP1 were co-transfected with the NanoBit eIF4E:eIF4G^604–646^ system with differing combinations of 4EBP1 mutant constructs with different sensitivities to mTORC1-mediated phosphorylation. Each transfection combination was normalised to its own DMSO treatment control. The molecular mass of the protein marker is indicated in kilodaltons

The results from the NanoBit assay demonstrated that both Torin and PP242 are much more potent inhibitors of eIF4F complex formation than Rapamycin in HEK293 cells (Fig. 2). The effect of Rapamycin on mTORC1 activity is mainly against weaker mTORC1 substrates, such as protein S6 kinase, whereas it has much more limited effect against better substrates such as 4EBP1 [42]. Western blot analysis demonstrated that Rapamycin, under the experimental conditions utilised by the NanoBit eIF4E:eIF4G^604–646^ system, was a much more efficient inhibitor of ribosomal S6 subunit phosphorylation, a direct target of the S6 kinase, than it was of total 4EBP1 phosphorylation in contrast to PP242 and Torin, which efficiently inhibited both (Fig. 3a, b). However, Rapamycin treatment does induce the rapid loss of the hyper-phosphorylated form of 4EBP1 (Fig. 3b) at low nanomolar concentrations, but this effect is insufficient to lead to disruption of the eIF4F complex, as demonstrated by its lack of potency in the NanoBit assay (Fig. 2c) and the corresponding m^7^GTP pulldown of the eIF4F complex (Fig. 2d). The dual MTORC1/2 inhibitors, PP242 and Torin, also led to a decrease in phosphorylation of AKT at position 473, the activated form of the kinase, which is directly regulated by mTORC2 as part of a positive feedback loop (Fig. 3a). Additionally, the IC_50_ value derived from the NanoBit system correlated well with the concentrations at which phosphorylation levels of 4EBP1 were abolished for each compound. These results demonstrate the NanoBit eIF4E:eIF4G^604–646^ system, combined with assays measuring other cellular outputs of inhibited proteins (i.e. mTOR activation of S6 kinase), can delineate the overlapping effects of different small molecules.

mTOR inhibitors ability to attenuate eIF4F formation should be strictly dependent on the available cellular pool of de-phosphorylated 4EBP1. Therefore, the NanoBit eIF4E:eIF4G^604–646^ system was transfected into PANC cells where the expression of 4EBP1 is at much lower levels than in HEK293 cells (Additional file 3: Figure S3A) [43]. PANC cells were then treated with PP2442, where the derived IC_50_ (0.07 ± 0.04 μM) as expected was significantly attenuated in comparison to NanoBit eIF4E:eIF4G^604–646^-transfected HEK293 cells (9.18 ± 4.25 μM). PP242 activity was restored in PANC cells by transfecting them with a mammalian vector expressing 4EBP1^WT^ (Fig. 3c). Corresponding experiments with 4EBP1^YLM^, which is unable to bind eIF4E and 4EBP1^4ala^ and thus is insensitive to mTOR inhibition, did not restore activity. Taken together, these results confirm the exquisite sensitivity of the NanoBit eIF4E:eIF4G^604–646^ to the release of endogenous eIF4E binding competent 4EBP1 and verifies its applicability as tool to test compounds phenotypically for eIF4F disruption.

### eIF4E S209 phosphorylation minimally affects eIF4F complex stability

**Fig. 4:**
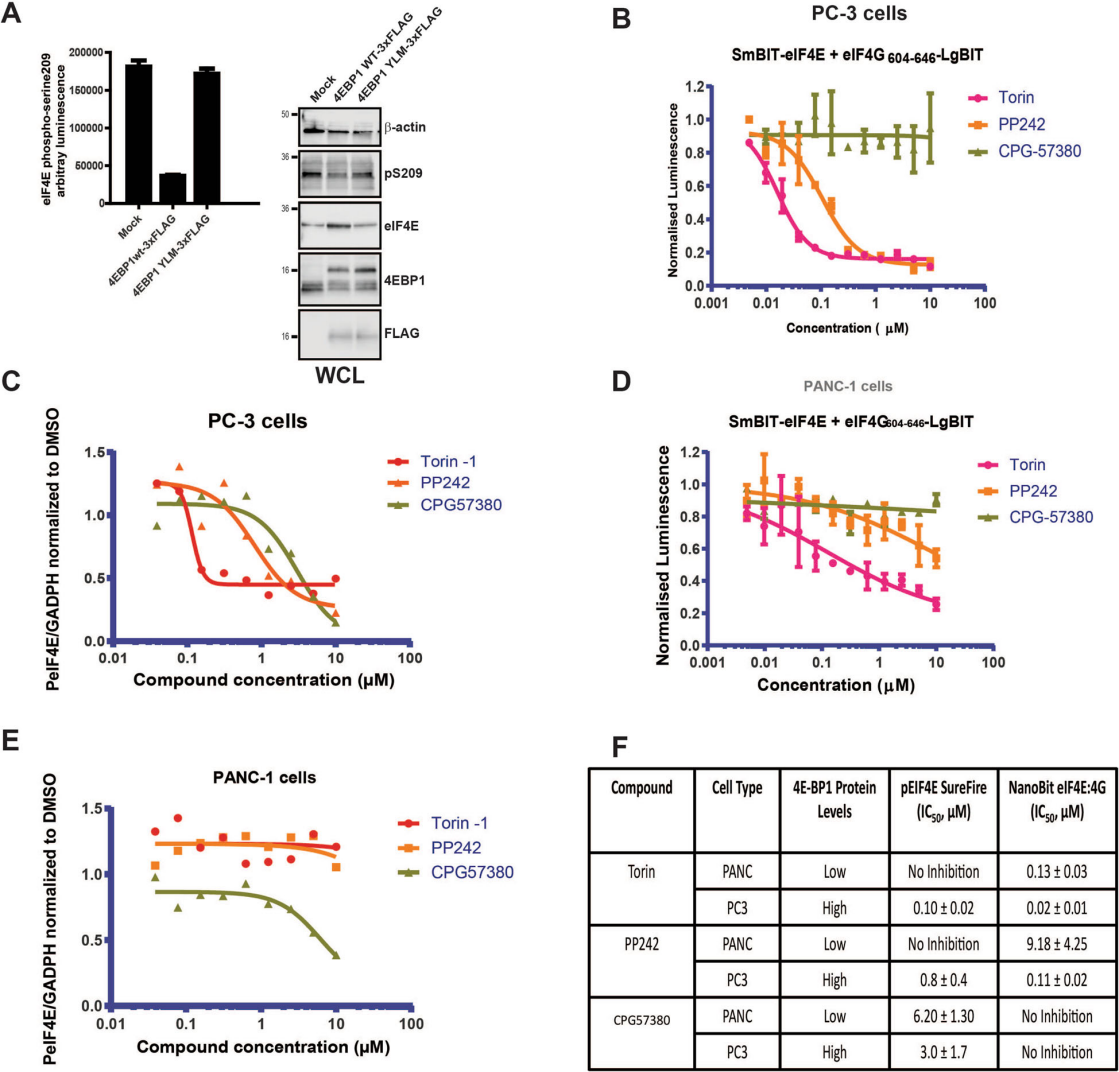
**a** HEK293 cells were transfected with either MOCK (empty vector), 4EBP1 WT (wild-type) or negative control 4EBP1 YLM construct. Cells were analysed 48 h post transfection for eIF4E^S209^ phosphorylation using the AlphaScreen Surefire assay. 4EBP1 constructs were also FLAG tagged. Inset western blot analysis of whole cell lysate derived from equivalent HEK293 cell transfections probed for phosphorylated eIF4E, endogenous 4EBP1, FLAG-tagged constructs and β-actin**. b** PC-3 cells transfected with the NanoBit eIF4E:eIF4G^606–646^ system and treated with compound titrations of either the mTORC1/2 active site inhibitors, Torin and PP242, or the MNK1/2 kinase inhibitor CGP57380. **c** PC-3 cells were treated with titrations of either Torin, PP242 or CGP57380, and eIF4E phosphorylation was determined using the AlphaScreen Surefire eIF4E^209^ phosphorylation assay. The signal was normalised to GAPDH levels, which were also determined using an AlphaScreen assay. **d** PANC-1 cells transfected with the NanoBit eIF4E:eIF4G^606–646^ system and treated with compound titrations of either the mTORC1/2 active site inhibitors, Torin and PP242, or the MNK1/2 kinase inhibitor CGP57380. **e** PANC-1 cells were treated with titrations of either Torin, PP242 or CGP57380, and eIF4E phosphorylation was determined using the AlphaScreen Surefire eIF4E^209^ phosphorylation assay. The signal was normalised to GAPDH levels, which were also determined using an AlphaScreen assay. **f** Table containing IC_50_ values determined for eIF4E S209 phosphorylation and NanoBit eIF4E:4G disruption. IC_50_ values were derived from fitting the relevant titration curves with four parameter models using non-linear regression (Prism, GraphPad Ltd.). All values represent mean ± SD (*n* = 3). The molecular mass of the protein marker is indicated in kilodaltons

eIF4E is also directly phosphorylated at Ser209 by MNK kinases 1/2, via their recruitment to the eIF4F complex through the MNK binding domain found in eIF4G. The MNK kinases themselves are regulated by the RAS/ERK and p38 MAPK pathways and are direct substrates of the ERK1/2 kinases. Western blotting confirmed that the ratio of phosphorylated SmBiT-eIF4E to non-phosphorylated SmBiT-eIF4E was at the same ratio as that observed for endogenous eIF4E protein (Additional file 3: Figure S3B), and that it was also sensitive to the MNK kinase inhibitor CGPG57380. It has been reported that disruption of eIF4E:eIF4G binding leads to a decrease in S209 phosphorylation by Mnk1/2 [43], as it can no longer be brought into close proximity by full-length eIF4G to eIF4E. Co-transfection experiments were performed with 4EBP1^4ALA^ and 4EBP1^YLM^ in the absence of kinase inhibition in HEK293 cells (Fig. 4a), which demonstrated that the disruption of the eIF4F complex specifically leads to eIF4E de-phosphorylation. Measurement of eIF4E phosphorylation with the AlphaScreen Surefire assay (Perkin-Elmer) was confirmed via western blot analysis (Fig. 4a).

To test the effect of S209 phosphorylation on eIF4F complex stability, the MNK kinase inhibitor CGP57380 was used to treat NanoBit eIF4E:eIF4G^604–646^ co-transfected PC-3 cells, whereupon no decrease in the luminescence signal or decrease in cell viability was measured, demonstrating that de-phosphorylation of SmBiT-eIF4E at position 209 has no effect on its interaction with the eIF4G^604–646^-LgBiT component of the NanoBit system in contrast to the mTORC1/2 inhibitors Torin and PP242 (Fig. 4b, Additional file 3: Figure S3C). PC-3 cells were also treated with either CGP57380, Torin or PP242 and monitored for MNK-mediated phosphorylation of eIF4E. Surprisingly, PP242 and Torin were significantly more potent at attenuating eIF4E S209 phosphorylation than the CGP57380 (Fig. 4c) inhibitor. The inhibition of phosphorylation at S209 by Torin and PP242 occurred under the concentration range where both inhibitors induced eIF4E:4G disruption (Fig. 4b, f), confirming the role of eIF4F complex formation in MNK-mediated phosphorylation of eIF4E. Interestingly, the ability of PP242 and Torin to disrupt the eIF4E: eIF4G^604–646^ PPI in the NanoBit system and to inhibit eIF4E phosphorylation was dramatically reduced in PANC1 cells, whilst CGP57380, which was still ineffective in disrupting the eIF4E:4G NanoBit system, only experienced a twofold decrease in its ability to prevent phosphorylation of eIF4E (Fig. 4d, e, f and Additional file 3: Figure S3D).

Taken together, these results show that PP242 and Torin do not have any off-target activity against Mnk1/2 kinases and that their effect on eIF4E phosphorylation is only mediated by 4EBP proteins. These experiments confirm that cells, which have a high or normal abundance of 4EBP1 and where un-phosphorylated 4EBP1 is readily available (i.e. upon mTOR1 inhibition), induce rapid eIF4E dephosphorylation by eIF4E:4G complex disruption, preventing eIF4G-MNK sub-complex mediated phosphorylation of eIF4E.

### Combined inhibition of RAS/ERK and PI3K/AKT signalling pathways additively disrupt eIF4F complex formation

**Fig. 5:**
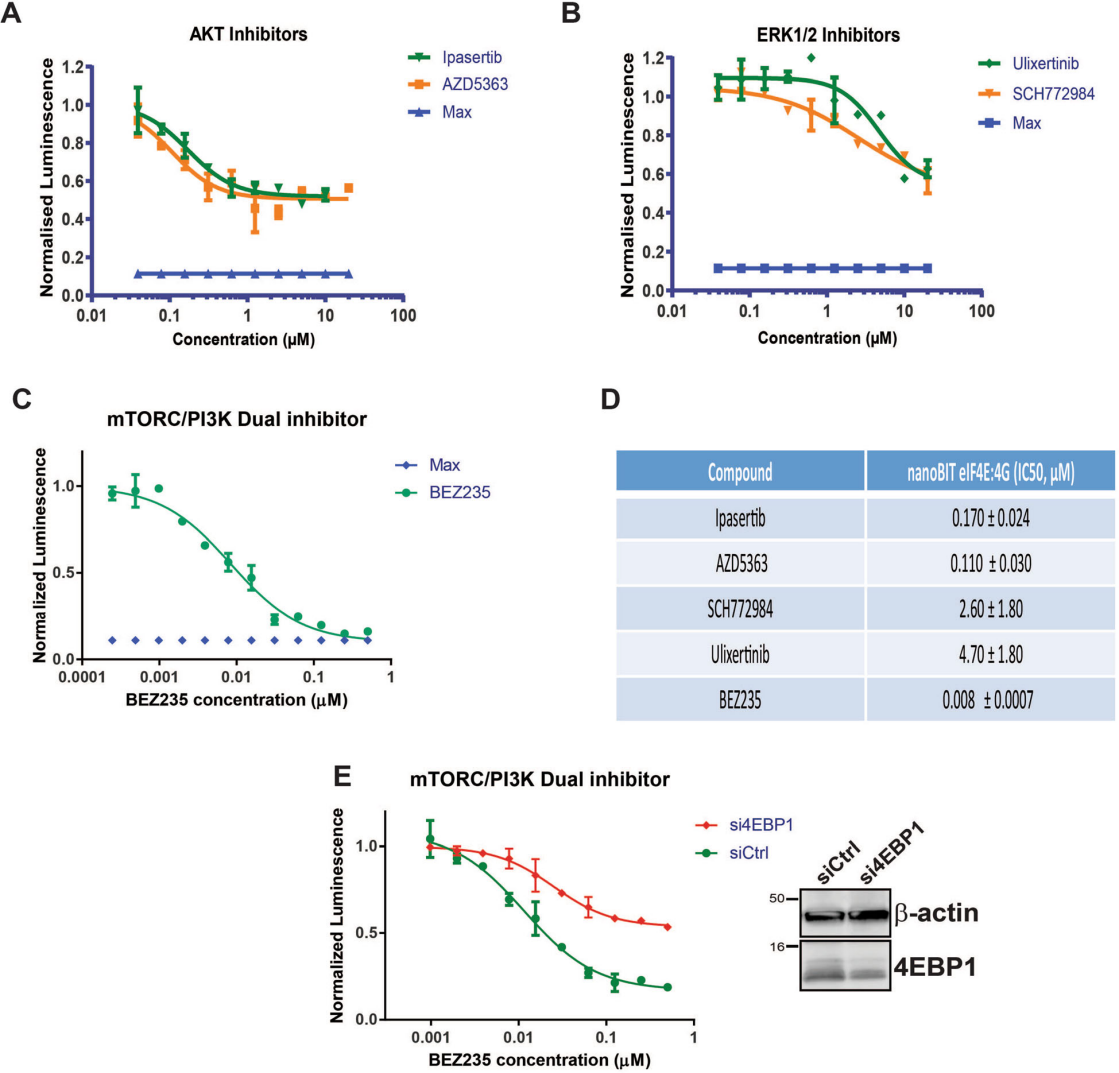
HEK293 cells transfected with the NanoBit eIF4E:eIF4G^604–646^ system and treated with compound titrations of either **a** the AKT inhibitors, Ipasertib and AZD5363, **b** the ERK1/2 inhibitors, Ulixertinib and SCH772984 or **c** the mTORC/PI3K dual inhibitor BEZ235 for 4 h, and then measured for luciferase activity. **d** Table displaying the IC_50_ values determined for each compound titrated into the live cell NanoBit eIF4E:eIF4G^604–646^ PPI system. **e** HEK293 cells were co-transfected with NanoBit eIF4E:eIF4G^604–646^ system and either siRNA Ctrl or siRNA 4EBP1. Cells were then treated BEZ235 and luciferase activity assessed as in **a**, **b** and **c**. Four parameter models were fitted to the experimental data using non-linear regression to derive the IC_50_ values (Prism, GraphPad Ltd.). All values represent mean ± SD (*n* = 3). Data was normalised to DMSO controls

The mTOR/4EBP1 axis serves to integrate differential signals from multiple growth factors interacting with their cognate receptor tyrosine kinases via the RAS/ERK and PI3K/AKT pathways to modulate cap-dependent translation via eIF4F complex assembly and eIF4E phosphorylation. Titrations were then performed in NanoBit eIF4E:eIF4G^604–646^ system with specific inhibitors against both ERK and AKT kinases. These kinases are critical transducers of the RAS/ERK and PI3K/AKT signalling pathways, respectively. Inhibition of both kinases gave sigmoidal inhibition profiles in the NanoBit eIF4E:eIF4G^606–646^ assay with the AKT-specific compounds exhibiting IC_50_s at 0.17 ± 0.02 μM and 0.11 ± 0.03 μM, whilst the ERK inhibitors possessed higher IC_50_s at 2.60 ± 1.80 μM and 4.70 ± 1.80 μM, respectively (Fig. 5a, b and d). However neither set of inhibitors induced complete disruption of the eIF4F complex as defined by the maximal dose of the mTORC1/2 dual inhibitor Torin (Fig. 5a, b). These results indicate that these pathways either regulate eIF4F complex formation in parallel to each or that another eIF4F regulating pathway is active and that both pathways affect eIF4F formation through the same mechanism. The compound BEZ235, a highly potent pan kinase inhibitor of mTORC1/2 and PI3K and its isoforms, was also tested in the NanoBit eIF4E:eIF4G^604–646^ system. In contrast to the AKT and ERK compounds tested, BEZ235 achieved maximal inhibition of eIF4F activity in relation to the control Torin treatment. Additionally, BEZ235 was determined to have an apparent IC_50_ of 8.00 ± 0.7 nM (Fig. 5c, d), which is substantially more potent that the mTORC1/2-specific inhibitors Torin and PP242 (Fig. 2a–c). This increase in potency also correlated with more efficient disruption of the eIF4E-eIF4G complex (Additional file 4: Figure S4A). As previously demonstrated with PP242 (Fig. 2f), BEZ235 activity was also substantially reduced by siRNA-mediated knockdown of 4EBP1 protein levels (Fig. 5e).

**Fig. 6:**
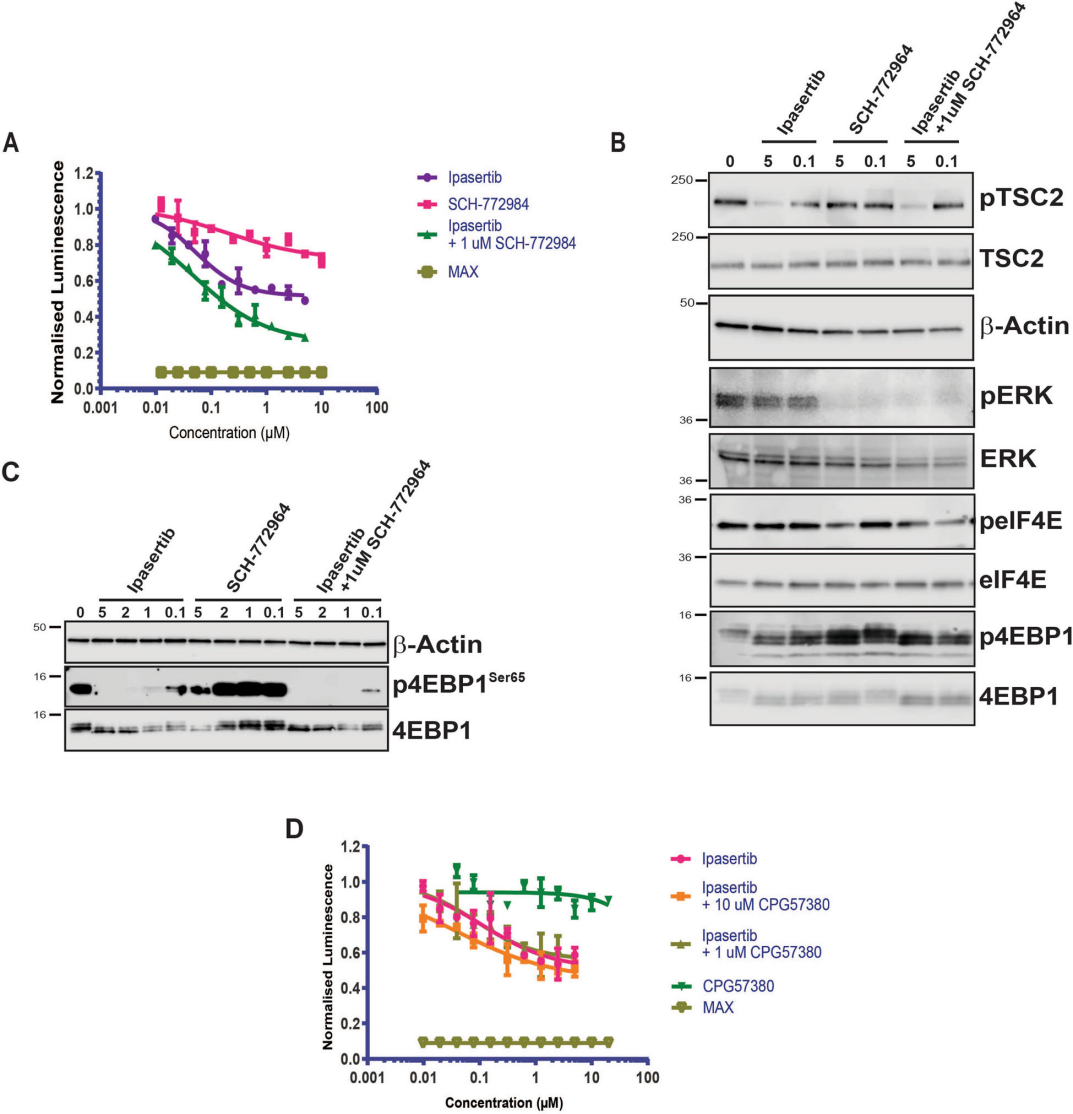
**a** HEK293 cells were transfected with the NanoBit eIF4E:eIF4G^606–646^ system and treated either with titrations of the AKT inhibitor Ipasertib or the ERK 1/2 inhibitor SCH-772984. Additionally, transfected HEK293 cells were also treated with a titration of Ipasertib with SCH772984 present throughput at constant concentration of 1 μM. Data was normalised to DMSO controls and equivalent cell viability experiments. **b** Western blot analysis of selected titration points in **a** probed for TSC2, ERK, eIF4E^S209^ and 4EBP1 phosphorylation status (T37/T46). **c** Western blot analysis of a wider range of titration points in **a** using anti-phospho Serine 65 4EBP1 antibody. Beta-actin was used as loading control. See “Materials and methods”. **d** Transfected HEK293 cells were treated with either titrations of Ipasertib, the MNK 1/2 inhibitor CGP57380, or with titrations of Ipasertib with CGP57380 kept at different constant concentrations. Treatments were performed for 4 h and then transfected HEK293 cells were assayed for luciferase activity. All values represent mean ± SD (*n* = 3). The molecular mass of the protein marker is indicated in kilodaltons

It has been reported that both AKT and ERK1/2 kinases negatively regulate 4EBP1 phosphorylation through parallel mechanisms to promote mTORC1 signalling [6, 44]. They principally affect 4EBP1 phosphorylation by regulating the activity of the TSC1/2 complex via phosphorylation events at distinct sites in TSC2, which in turn controls the activity of mTORC1 via RHEB [45, 46]. Using the NanoBit eIF4E:eIF4G^604–646^ system, we have already been shown that specific inhibition of either pathway alone only leads to partial antagonism of eIF4F complex formation. Both pathways were therefore inhibited together with combined Ipasertib and SCH-772984 treatment to examine for potential synergetic or additive modes of action on eIF4F activity. Several titrations of Ipasertib were performed in the NanoBit eIF4E:eIF4G^604–646^ with increasing concentrations of SCH-772984, which clearly demonstrated that SCH-772984 at 1 μM had an additive effect on eIF4F disruption by liberation of 4EBP1 from phosphorylation (Fig. 6a). Western blot analysis further demonstrated that AKT kinase inhibition lead to decreased phosphorylation of TSC2 at a site independent from ERK-mediated phosphorylation (Fig. 6b). Although we failed to detect ERK mediated-phosphorylation of TSC2 directly, inhibition of ERK was occurring as shown by the decrease in ERK auto-phosphorylation (Fig. 6b). Both ERK and AKT inhibition lead to 4EBP de-phosphorylation with the AKT inhibitor Ipasertib proving to be more efficient at the concentrations tested than the ERK inhibitor SCH-772964 (Fig. 6b, c). These results correlated with their differing potencies in the NanoBit PPI assay. Combined inhibition of the AKT and ERK kinases leads to greater dephosphorylation of 4EBP1 as confirmed by the more intense staining of the more electrophoretically mobile 4EBP1 species and by specific de-phosphorylation at serine 65 (Fig. 6b, c). In agreement with this data, siRNA-mediated 4EBP1 knockdown attenuated the inhibitory effects of Ipasertib and the additive effects of the combination treatment observed in the NanoBit eIF4E:eIF4G^604–646^ system (Additional file 4: Figure S4B and-S4C). The effects of 4EBP1 knockdown were more difficult to discern for SCH-772964 in the NanoBit system. However, SCH-772964 is only observed to induce serine 65 de-phosphorylation of 4EBP1 (Fig. 6b) and total 4EBP1 dephosphorylation (Fig. 6b) at 5 μM. This may indicate that the additive effects induced by SCH-772944 at 1 μM when in combination with Ipasertib are not being directed through 4EBP1. These results demonstrate the role of 4EBP1 in mediating the effects of Ipasertib alone and as part of the combination treatment. Additionally, they also suggest that SCH-772964 may be having ERK-independent effects or that the ERK kinases may be interfering with eIF4F complex via an alternative mechanism. Further experiments would be required to understand these effects.

ERK1/2 kinases as well as regulating the activity of the TSC1/2 complex are also responsible for controlling the activation of the MNK1/2 kinases that mediate eIF4E phosphorylation at position S209. HEK293 cells when treated with Ipasertib and CGP57380, a specific inhibitor of MNK1 and MNK2, demonstrated that AKT inhibition combined with inhibition of eIF4E S209 phosphorylation did not lead to a larger decrease of the luminescence signal in the NanoBit eIF4E:eIF4G^604–646^ assay than with Ipasertib alone (Fig. 6d). These results support reports that the RAS/ERK pathway affects eIF4F complex formation via crosstalk with the PI3K/AKT pathway rather than by directly regulating the phosphorylation status of eIF4E [6, 44].

### Abundance of de-phosphorylated 4EBP1 available to disrupt the eIF4F complex dictates drug resistance

**Fig. 7:**
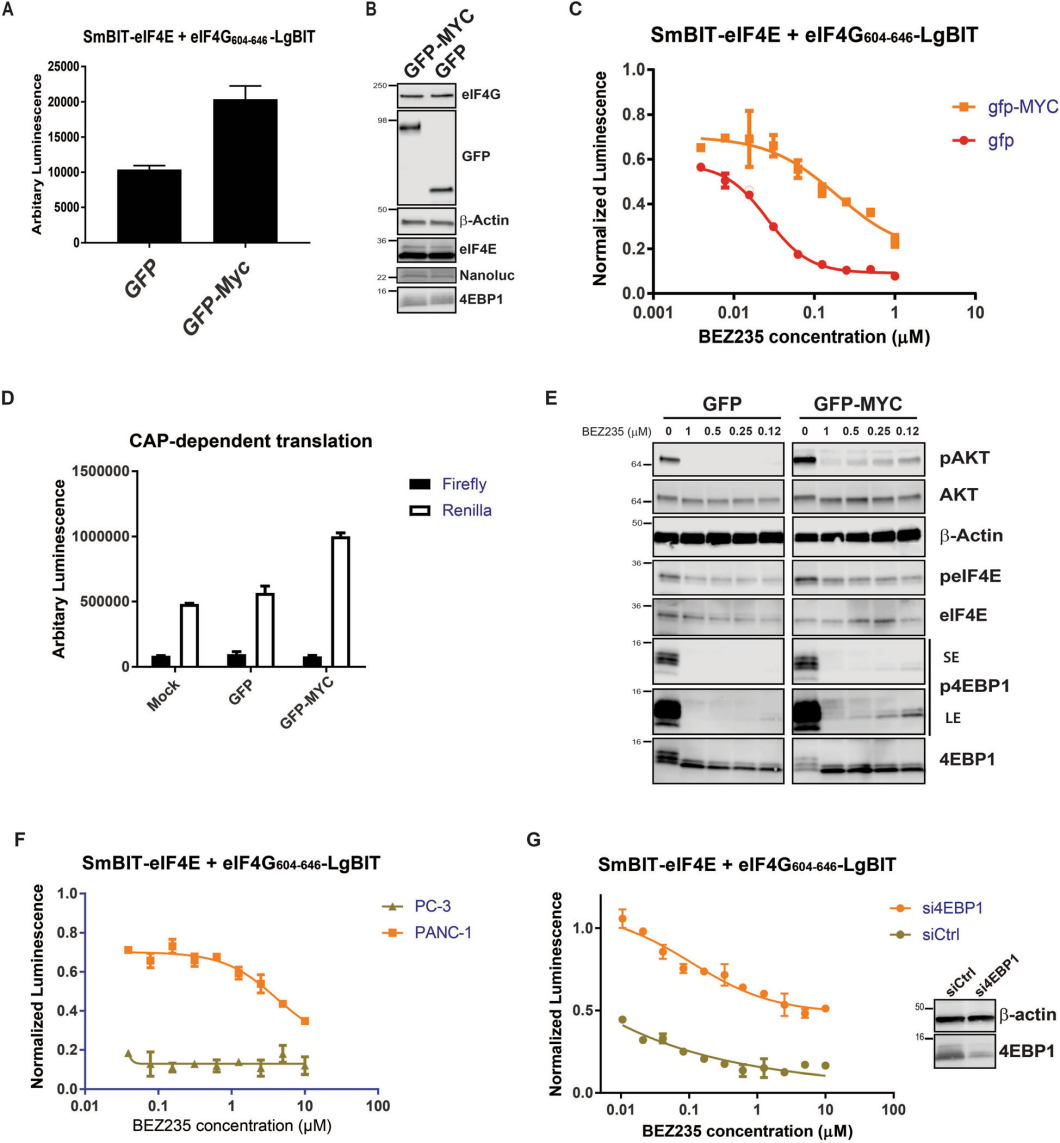
**a** Basal NanoBit eIF4E:eIF4G^606–646^ luciferase activity in cells both co-transfected with GFP or GFP-Myc and the NanoBit eIF4E:eIF4G^606–646^ system. **b** Western blot analysis of HEK293 cells co-transfected with the NanoBit eIF4E:eIF4G^606–646^ system and either GFP or GFP-MYC post 48 h transfection. **c** HEK293 cells were co-transfected with the NanoBit eIF4E:eIF4G^606–646^ system with either GFP or GFP-MYC. 48 h post-transfection HEK293 cells were titrated with BEZ235 and then assayed for luciferase after 4 h after treatment. **d** A bicistronic luciferase reporter, which measures the relative amount of cap-dependent translation (Renilla) to cap-independent translation (Firefly), was co-transfected with either empty vector (MOCK), GFP or GFP-Myc into HEK293 cells. Renilla and Firefly luciferase activity was measured 48 h post transfection and plotted as a ratio-metric value. The Renilla luciferase is under the control of a GC-rich 5′UTR whose activity correlated to cap-dependent translation, whilst the Firefly luciferase is regulated by an internal ribosomal site (IRES). **e** Western blot analysis of the BEZ235 titrations shown in **c** performed under identical co-transfection and treatment conditions (SE = short exposure, LE = long exposure). **f** Comparative plot of PANC1 and PC-3 cells co-transfected with the NanoBit eIF4E:eIF4G^606–646^ system and treated with the dual mTOR/PI3K inhibitor BEZ235. Cells were assayed 4 h post treatment. **g** After 48 h co-transfection with NanoBit eIF4E:eIF4G^606–646^ system and siRNA Ctrl or siRNA 4EBP1, PC-3 cells were treated with BEZ235 as for **f** and then assayed for luciferase activity. The level of 4EBP1 siRNA knockdown was demonstrated by western blot using anti-4EBP1 antibody. Beta-actin was visualised as a loading control. All values represent mean ± SD (*n* = 3). The molecular mass of the protein marker is indicated in kilodaltons

Several studies have demonstrated that the activity of the eIF4F complex is a central driver in resistance against various therapies used to treat cancer [24, 25]. It had been demonstrated that c-Myc overexpression mediates drug resistance against the PI3K/AKT/mTORC1/2 inhibitor BEZ235 by promoting eIF4F complex activity [24]. To assess the capability of the NanoBit eIF4E:eIF4G^604–646^ system to measure the effect of anti-cancer drug resistance mechanisms on the eIF4F complex status, the effect of MYC overexpression on the potency of BEZ235 was tested by co-transfecting GFP fused v-Myc (referred to as GFP-MYC from now on) alongside the components of the protein complementation system. Viral Myc was used as it is a more potent transformer of cells than c-Myc, and we thus hypothesised it would have more profound effects on chemo-resistance to BEZ235 [47]. The basal NanoBit signal in the GFP-MYC-transfected cells was higher by a factor of two than that in the GFP control-transfected HEK293 cells (Fig. 7a). Western blot analysis demonstrated that there was no dramatic change in the expression of the two components that formed the NanoBit eIF4E:eIF4G^604–646^ complex (Fig. 7b). This result implied that the increase in the NanoBit signal is a product of more efficient complex formation. Overexpression of GFP-MYC in the NanoBit system significantly decreased the potency of BEZ235 by over an order of magnitude (Fig. 7c). This reduction in potency was not observed when the identical experiment was performed with GFP vector only control.

The effect of GFP-MYC cap-dependent translation was also assessed using a bicistronic luciferase reporter, which measures the relative amounts of cap-dependent translation to cap-independent (internal ribosomal entry site (IRES) dependent) translation. The GFP-MYC-transfected cells demonstrated significantly elevated levels of cap-dependent translation versus the GFP non-fusion control (Fig. 7d). eIF4E protein levels and other components of the eIF4F complex are transcriptionally regulated by c-Myc via the presence of E-box repeats in their promoters [48]. However, when HEK293 cells transfected with either GFP-MYC or GFP control vector were probed for relative differences in expression of eIF4F components, no significant difference could be seen in their protein levels (Fig. 7b).

To understand the molecular basis for the v-Myc-driven increase in eIF4F activity measured in the NanoBit assay and resistance to BEZ235 treatment, the phosphorylation status of 4EBP1 was assessed across the titration curves for both sets of transfected HEK293 cells (Fig. 7e). This revealed that in the GFP-MYC-transfected cells treated with BEZ235, the population of phosphorylated 4EBP was more resistant to dual mTORC1/2 and PI3K inhibition. In contrast, in the cells transfected with GFP vector control, no residual phosphorylated 4EBP1 could be detected upon BEZ235 treatment. The amount of phosphorylated AKT was also significantly higher in the v-Myc overexpressing cell population, indicating that the levels of active mTORC1/2 are also significantly greater. This hyper-activation of mTORC1/2 is sufficient to allow enough 4EBP1 to remain phosphorylated, which enables residual eIF4F activity upon BEZ235 treatment and in turn maintenance of cap-dependent translation and chemo-resistance. Additionally, western blot analysis demonstrated higher levels of phosphorylated eIF4E in the v-Myc overexpressing cells, further evidence for increased eIF4F complex formation (Fig. 7e). The effect of BEZ235 in the NanoBit eIF4E:eIF4G^604–646^ system was also measured using PC3 and PANC cells, where the expression of endogenous 4EBP1 is at much lower levels than in PC3 or HEK293 cells (Fig. 7f). The activity of BEZ235, as was observed in the v-Myc overexpressing HEK293 cells, was dramatically attenuated in PANC cells, whilst in PC-3 cells, where normal levels of 4EBP1 are expressed and where de-phosphorylation can readily occur, BEZ235 once again demonstrated activity in the mid-nanomolar range. Consistently with this and previously obtained results (Fig. 5e), siRNA 4EBP1 knockdown strongly attenuated BEZ235 activity in PC3 cells (Fig. 7g) further demonstrating that the availability of un-phosphorylated 4EBP1 is responsible for BEZ235 drug efficacy.

## Discussion

The eIF4F complex is frequently dysregulated in human tumours due to activating mutations in critical genes in the PI3K/AKT and RAS/ERK signalling pathways that regulate its formation [1, 2]. These two signalling pathways principally regulate eIF4F activity, via negative regulation of 4EBP1 binding to eIF4E through mTORC1-mediated phosphorylation at critical residues (T37, T46, S65 and T70) [15]. 4EBP1 when not phosphorylated at these positions binds eIF4E and competitively displaces eIF4G from it, resulting in eIF4F complex disruption and downregulation of cap-dependent translation. However, these pathways regulate multiple other targets and processes as well such as cell cycle progression, transcription and apoptosis [49, 50]. In this paper, we have developed an eIF4E:eIF4G NanoBit biosensor that accurately measures the disruption of the eIF4F complex by endogenous 4EBP1 upon mTORC1 inhibition and allows us to delineate specific effects of small molecule inhibitors on the eIF4F complex. Dual inhibition of mTORC1/2 by small molecule kinase antagonists, such as PP242 [41] and Torin [40], was shown to be significantly more potent than rapalogs, allosteric inhibitors of mTORC1 functions, in inducing dephosphorylation of 4EBP1.

The eIF4E:eIF4G NanoBit biosensor unlike other reported assays in the literature that measure eIF4F complex disruption, such as an in vitro TR-FRET (time-resolved – fluorescence excitation transfer)-based method [35] that relies on purified protein and a PLA (proximity ligation assay) [34] methodology that measures the integrity of endogenous eIF4F complex in fixed and permeabilised cells, can quantify eIF4F complex integrity in live cells. This is advantageous as it allows the cellular permeability of compounds to be assessed alongside disruption of the target of interest, as well as decreasing the number of experimental steps required in processing the samples. Measuring the activity of the complex in live cells allows any artefacts associated with fixation and permeabilisation to be minimised, such as the release of impermeable compounds entrapped in endosomes or bound on the extracellular surfaces of cell membranes, which could give rise to false positive readings for compounds that would otherwise not enter the cell interior. This is especially critical in the pursuit of new modalities that are being developed to target intracellular targets that are intractable to small molecule inhibition, as majority of these compounds are inherently cell impermeable, and thus assays that accurately measure intracellular activity of novel compounds are required to develop robust structure activity relationships. Both the PLA and NanoBit technologies also enable, unlike the in vitro TR-FET assay, the analysis of regulatory pathway perturbation on the eIF4F complex.

Using small molecule inhibitors specific for ERK or AKT, the regulation of the eIF4F complex by the RAS/ERK and PI3K/AKT pathways could be conclusively shown to be independent of S209 phosphorylation by MNK1/2 kinases and to cause dephosphorylation of 4EBP1 at Ser65 (Fig. 6c). Inhibition of either the ERK or AKT kinases leads to a reproducible and significant inhibition of eIF4F activity as measured by the eIF4e:eIF4G biosensor (Fig. 5a, b). However, neither inhibitor alone could induce a similar decrease in the assay signal when compared to the dual mTORC1/2 inhibitors (Torin and PP242) indicating only partial disruption of the eIF4F complex. Interestingly, CGP57380 treatment, a specific MNK1/2 kinase inhibitor, led to no decrease in eIF4F activity (Figs. 4b, d and 6d), confirming that ERK regulation of eIF4F complex is occurring via an alternative mechanism and independent of S209 phosphorylation. These results suggest that 4EBP1 is acting as a signalling hub and integrating the signals from both pathways to control the activity of the eIF4F complex, agreeing with the conclusions made by She et al. [6] and Winter et al. [44], respectively. Combined inhibition of both ERK and AKT kinase by Ipasertib and SCH772984 leads to an additive effect on eIF4F disruption and 4EBP1 phosphorylation (Fig. 6a, b, c), further suggesting that both signalling pathways act independently on 4EBP1 phosphorylation. However, knockdown of 4EBP1 did not perturb SCH-772984 activity in HEK293 cells (Additional file 4: Figure S4B and S4C) thus suggesting that the effects of SCH-772984 were independent of 4EBP1 at this concentration. Winter et al. have postulated that differential regulation of distinct phosphorylation sites on the TSC1/2 complex by ERK and AKT regulate 4EBP1 phosphorylation via mTORC1 [44]. Our studies add to evidence that direct phosphorylation of eIF4E at S209 does not affect the stability of the eIF4F complex itself. However, multiple mouse studies have demonstrated that phosphorylated eIF4E is involved in increasing the translation rates of a subset of mRNAs that play a significant role in tumour development. The exact molecular role of phosphorylated eIF4E in cap-dependent translation still remains unclear. However, in contrast, eIF4F complex formation plays a critical role in eIF4E phosphorylation, where MNK1/2 is recruited to the complex by eIF4G allowing phosphorylation to occur. Increases in the amount of 4EBP1 competent to interact with eIF4E either by expression of exogenous 4EBP1^4ALA^ or by mTORC1 antagonism, lead to eIF4E de-phosphorylation by preventing binding of eIF4G to eIF4E.

The dual PI3K/mTOR inhibitor BEZ235 was also characterised in the eIF4E:4G NanoBit assay and was shown out of all the compounds tested to be the most potent in decreasing eIF4F abundance, with an IC_50_ substantially lower than those derived for PP242 and Torin. It has been increasingly clear from reports in the literature that the activity of the eIF4F complex contributes to resistance development against many types of therapies used in cancer treatment, e.g. Vermurafenib (B-RAF (V600E) inhibitor) [34], AZD8055 (mTORC1/2 inhibitor) [25] and receptor tyrosine kinase inhibitors such as erlotinib and cetuximab [51]. Interestingly, c-Myc and eIF4E overexpression have been shown to promote resistance to BEZ235 in immortalised mammary epithelial cells [24, 25]. Using the eIF4E:4G NanoBit system, we demonstrated that overexpression of v-Myc results in a dramatic attenuation of the ability of BEZ235 to disrupt the eIF4F complex HEK293 cells. v-Myc was utilised rather than c-Myc as it is more efficient in transforming cells, and we hypothesised that it would have a greater effect in terms of chemo-resistance [47]. Contrary to our expectations, we did not observe v-Myc overexpression significantly increasing the protein levels of two core components of the eIF4F complex, namely eIF4E and 4G (Fig. 7b), which have been reported to be transcriptionally controlled by c-Myc [48]. However, a corresponding increase in a cap-dependent translation using a bicistronic assay did occur. Interestingly, our results revealed that in cells overexpressing v-Myc, BEZ235 did not induce 4EBP1 dephosphorylation as efficiently. Therefore, it seems that v-Myc overexpression has led to the development of a 4EBP1 population that is resistant to de-phosphorylation by inhibition of mTORC1, thus allowing the eIF4F complex to be maintained and remain translationally active. Analysis of the western blot (Fig. 7e) suggests this is principally driven by v-Myc-driven hyper-activation of mTORC1 via the AKT signalling pathway. Reports have been published describing the positive effects of c-Myc overexpression on mTORC1 function [48]. The chemo-resistance observed in GFP-Myc overexpressing HEK293 cells is intuitively similar to the lack of activity of BEZ235 in PANC1 cells, where endogenous levels of 4EBP1 are very low and thus prevent de-phosphorylated 4EBP1 from accumulating to the level to prevent eIF4F complex formation. These results suggest that it is the inability of cells to increase the relative abundance of dephosphorylated 4EBP1 to eIF4E levels that drive eIF4F-mediated resistance mechanisms upon drug treatment. These findings highlight the importance of discovering highly efficacious direct inhibitors of eIF4E and eIF4F assembly.

## Conclusions

The results in this paper demonstrate that a large proportion of the compounds that target the RAS/ERK and PI3K/AKT pathways and the mTOR complexes decrease eIF4F complex formation to differing degrees, and that specific inhibition of RAS and ERK kinases only leads to partial disruption of the eIF4F complex due to both pathways regulating mTORC1 through distinct mechanisms. This confirms that direct inhibition of eIF4F activity is a therapeutic valid approach as has been suggested by various animal models [52, 53]. In addition, factors that modulate eIF4F activity through regulation of the relative abundance and phosphorylation status of 4EBP1 and the expression of the constituent components of eIF4F are emerging as a common mechanism that drive resistance to a large variety of drugs, which further support the attractiveness of eIF4F and its components as therapeutic targets. We envisage that the NanoBit eIF4E:4G live cell PPI assay will be vital in discovering novel compounds (e.g. macrocyclics, constrained peptides, natural products) that can efficiently disrupt formation of the eIF4F complex and in optimising their delivery into the cell.

## Materials and methods

### Plasmid and reagents

All eIF4E, eIF4G (including the Y624A, L629A and L630A binding mutant) and 4EBP1 mutant cDNAs were synthetized and obtained from IDT (Integrated DNA Technologies). eIF4E and eIF4G^604–646^ were cloned into NanoBit plasmids using the NanoBit PPI starter system (Promega) using XhoI/EcoRI and NheI/EcoRI cloning sites respectively. 4EBP1 mutants were cloned into pCDNA3.1 vector DNA (Thermo Fisher Scientific) harbouring a C-terminal 3× FLAG tag via NheI/BamHI sites to allow mammalian cell overexpression. For bacterial expression, 4EBP1 mutants were cloned into pGEX6P1 using BamHI/EagI cloning sites. GFP and v-Myc coding sequence residing in a pCMV6 mammalian expression vector were obtained from Origene. The bicistronic luciferase reporter construct pcDNA3-rLuc-polIRES-fLuc was purchased from Addgene. PP242, Torin1, Rapamycin, 4EGi-1 and 4E1RCat were purchased from Tocris Bioscience, whilst all other chemicals unless otherwise stated were purchased from Selleck Chemicals. siRNAs targeting either 4EBP1 (ON-TargetPlus Human EI4EBP1, J-003005-12-0005) or non-targeting control (ON-TargetPlus Non-targeting pool, D-001810-10-05) were purchased from DHARMACON.

### Protein purifications

eIF4E and 4EBP1 mutants were cloned into the GST fusion expression vector pGEX-6P1 (GE Lifesciences). BL21 DE3 competent bacteria were then transformed with the GST-tagged fusion constructs. A single colony was picked and transformed cells were grown in LB medium at 37 °C to an OD600 of ~ 0.6 and induction was carried out overnight with 0.3 mM IPTG at 16 °C. Cells were harvested by centrifugation, and the cell pellets were resuspended in PBS (Phosphate Buffered Saline, 2.7 mM KCl and 137 mM NaCl, pH 7.4) and then sonicated. The sonicated sample was centrifuged for 60 min at 17,000*g* at 4 °C. The supernatant was applied to a 5 ml FF GST column (Amersham) pre-quilibrated in PBS buffer with 1 mM DTT. The column was then further washed by 6 volumes of PBS. Proteins were then purified from the column by cleavage with PreScission (GE Lifesciences) protease. Ten units of PreScission protease, in one column volume of PBS with 1 mM DTT buffer, were injected onto the column. The cleavage reaction was allowed to proceed overnight at 4 °C. The cleaved protein was then eluted off the column with wash buffer. Protein fractions were analysed with SDS page gel and concentrated using a Centricon (3.5 kDa MWCO) concentrator (Millipore). Protein samples were then dialyzed into a buffer solution containing 20 mM Tris pH 8.0 with 1 mM DTT and loaded onto a mono Q column pre-equilibrated in buffer A (20 mM Tris, pH 8.0, 1 mM DTT). The column was then washed in 6 column volumes of buffer A and bound protein was eluted with a linear gradient of 1 M NaCl over 25 column volumes. Protein fractions were analysed with SDS page gel and concentrated using a Centricon (3.5 kDa MWCO) concentrator (Millipore). The cleaved constructs were then purified to 90% purity. Protein concentration was determined using A280.

### Competitive fluorescence anisotropy assay and *K*_*d*_ determination

Apparent *K*_*d*_ values were determined for linear peptides and purified 4EBP mutants via competitive anisotropy experiments as described previously [32]. All titrations were carried out in triplicate. Curve fitting was carried out using Prism 4.0 (GraphPad).

### Preparation of compound stock and working solutions

Ten millimolars or 1 mM stock solutions of compounds were prepared in 100% DMSO. Each compound was then serially diluted in 100% DMSO and further diluted 10-fold into HPLC-grade sterile water to prepare 10× working solutions in 10% DMSO/water of each compound. Depending on the required volume used in the relevant assay, compounds were added to yield final concentrations as indicated in the relevant figure with a residual DMSO concentration of 1% *v*/*v*.

### Cell culturing conditions

HEK293 (Thermo Fisher Scientific) were cultured in DMEM cell media, whilst PANC-1 (ATCC) and PC-3 (ATCC) were cultured in F12 glutammax medium. Both cell media were supplemented with 10% foetal calf serum (FBS) and penicillin/streptomycin. All cell lines were maintained in a 37 °C humidified incubator with 5% CO_2_ atmosphere.

### NanoBit eIF4E:eIF4G^604–646^ construction and validation experiments

Opaque 96-well plates were seeded with 20,000 HEK293 cells per well in DMEM and 10% FCS. Transfections in 96-well plate format were performed using FUGENE6 (Roche) with 100 ng of the indicated plasmid vectors per well as outlined in the manufacturer’s instructions (Fig. 1). Twenty-four hours after transfection, the medium was replaced with 100 μl of Opti-MEM I cell media containing 0% FCS with no added red phenol (Thermo Fisher Scientific) and luminescence activity assayed (as outlined in “NanoBit and cell viability experiments” section). Transfections utilising 4EBP1 constructs were performed with either 50 ng of the indicated construct or empty vector.

### NanoBit and immunoprecipitation experiments

Twenty-four hours prior to transfection, cells (HEK293, PANC-1 or PC-3) were seeded at a cell density of 800,000 cells per well of a six-well plate (Thermo Fisher Scientific). Transfections in six-well plates were performed using Lipofectamine 3000 (Thermo Fisher Scientific) with 3 μg of the indicated plasmid vectors per well according to the manufacturer’s instructions. For knockdown experiment, siRNA Ctrl or 4EBP1 at a final concentration of 100 nM were included in the transfection mixture. Transfections utilising GFP and GFP-MYC contained 1 μg of each vector or empty vector, respectively. In the case of transfections utilising any of the 4EBP1 constructs described, only 0.5 μg of each vector or empty vector was used, respectively. After a 24- or 48-h (as indicated in the figure legends) incubation period, the medium was removed and cells were washed with PBS saline. Cells were then either lysed directly for immunoprecipitation experiments or were trypsinised and re-suspended in Opti-MEM media with 0% FCS to perform NanoBit and cell viability experiments in a 96-well format.

#### NanoBiT and cell viability experiments

Either HEK293, PC-3 or PANC-1 cells were spun down at 1000 rpm for 5 min at room temperature. Supernatant was then discarded, and cells re-suspended to a density of to 220,000 cells per millilitre in Opti-MEM I cell media containing 0% FBS with no added red phenol. Then 90 μl of the cell re-suspension was added to the wells of a white opaque 96-well plate. Compounds were then titrated onto the 96-well plate using a suitable twofold dilution series from a 10× stock solution containing 10% *v*/*v* DMSO. Control wells were also treated with a 10% DMSO only stock solution to yield a final residual DMSO concentration of 1% *v*/*v*. The 96-well plates were then incubated for 4 h at 37 °C, with 5% CO_2_. Wells were then treated with a solution of Nano-Glo live substrate, which was prepared as per the manufacturer’s instructions. The plates were then shaken for 1 min at 22 °C and luminescence assessed after additional 50 min using an Envision Multi-Plate reader (Perkin-Elmer). Plates were then multiplexed with CellTiter-GLO 2.0 reagent (PROMEGA) and cellular viability determined according to the manufacturer’s instruction. NanoBit titration data was used to determine IC_50_ values for drug potency by fitting 4 parameter logistic curves using GraphPad Prism 7.0.

#### Immunoprecipitation (IP) and m^7^GTP pulldown experiments

Cells were prepared in six-well plates (as described in the NanoBit and Immunoprecipitation Experiments section) and grown to confluence. Cells were washed with phosphate buffered saline and directly lysed with 300 μl of lysis buffer containing 20 mM Hepes pH 7.4, 100 mM NaCl, 5 mM MgCl_2_, 0.5% NP-40, 1 mM dithiothreitol, and protease (Roche) and phosphatase (Sigma-Aldrich) inhibitor cocktail sets added as outlined by the manufacturer’s protocols. Cellular debris was separated by centrifugation, and the protein concentration was then determined using the BCA system (Pierce). m^7^GTP pulldown and FLAG IP experiments were performed with 200 μg of cell lysate, which was either incubated with 20 μl of either m^7^GTP (Jena Bioscience) agarose beads or anti-FLAG M2 antibody (Roche) immobilised agarose beads for 2–4 h at 4 °C on a rotator. Beads were then washed four times with lysis buffer containing no protease or phosphatase inhibitors. This was then followed by the addition of Laemlee buffer (2×) and the beads boiled for 5 min at 95 °C. Samples were centrifuged and the supernatant removed for western blot analysis.

### Protein expression analysis

Either non-transfected or transfected HEK293 cells (prepared as described in the NanoBit and Immunoprecipitation Experiments sections) were seeded with 20,000 cells per well in 96-well plates in OPTI-MEM cell media containing no serum. Plates were incubated for 4 h to allow cell attachment and then treated with the indicated compound and concentration. Post 4 h treatment, cells were washed with PBS and directly lysed in the wells of the plate with 100 μl of cell lysis buffer (20 mM Hepes pH 7.4, 100 mM NaCl, 5 mM MgCl_2_, 0.5% NP-40, 1 mM dithiothreitol, and protease (Roche) and phosphatase (Sigma-Aldrich) inhibitor cocktail sets added as outlined by the manufacturer’s protocols. Cellular debris was separated by centrifugation, and the protein concentration was then determined using the BCA system (Pierce). Samples were then used for western blot analysis.

### Western blot analysis

Samples were resolved on Tris-Glycine 4–20% gradient gels (Bio-Rad) according to the manufacturer’s protocol. Western transfer was performed with an Immuno-blot PVDF membrane (Bio-Rad) using a Trans-Blot Turbo system (Bio-Rad). Western blots were then performed. Antibodies against GFP, peIF4E^S209^ and 4EBP1 were purchased from Abcam. Antibodies against eIF4E, p4EBP1^Thr37/46^, p4EBP1^Ser65^ eIF4G1, β-actin, TSC2, pTSC2 (Thr1462), p44/42(ERK)^Thr202/Tyr204^, p44/42(ERK), AKT, pAKT (Ser473), S6 and pS6 (Ser235–236) were all purchased from Cell Signalling Technology. FLAG and NanoLuc antibodies were bought from Sigma and Promega, respectively.

### Cap-dependent translation assay

Opaque 96-well plates were seeded with 20,000 HEK293 cells per well in DMEM and 10% FCS. Transfections in a 96-well plate format were performed using FUGENE6 (Roche) with 30 ng of the bicistronic reporter plasmid (pcDNA3-rLuc-polIRES-fLuc) [54] and 250 ng of the indicated plasmid (GFP and GFP-MYC). twenty-four hours after transfection, the medium was replaced with 100 μl of Opti-MEM I cell media containing 0% FCS with no added red phenol (Thermo Fisher Scientific). Renilla and firefly luminescence activity was determined 4 h later using the Dual Reporter Assay System (PROMEGA). Luminescence readings were performed using an Envision Multi-plate reader (PerkinElmer), equipped with an automated dispenser.

### AlphaScreen Surefire assay

Ninety-six-well plates were seeded at 30,000 cells per well with non-transfected PC-3, PANC-1 or HKEK293 cells in DMEM with 10% FCS and incubated overnight. Cells were then washed with PBS and the media replaced with OPTI-MEM containing no serum. Cells were then treated with the indicated compounds. Controls were performed with DMSO vehicle treatments. Four hours post treatments, cells were lysed with 50 μl of passive lysis buffer (PerkinElmer) for 15 min and 10 μl of lysate from each well was transferred into a white bottom 384-well plate. GAPDH and pS209 eIF4E levels were assessed according to the manufacturer’s instructions (PerkinElmer) for each sample. Luminescence was measured using an Envision Multiplate reader (PerkinElmer).

AlphaScreen Surefire analysis of cells transfected with 4EBP1 vector constructs were performed using 96-well plates seeded with 30,000 HEK293 cells per well. Transfections in a 96-well plate format were performed using FUGENE6 (Roche) with 100 ng of the indicated plasmid vectors per well as outlined in the manufacturer’s instructions. GAPDH and pS209 eIF4E levels were assessed as described in the previous paragraph.

## Additional files

Additional file 1: **Figure S1.** (A) eIF4E and SmBIT-eIF4E (indicated with black arrows), eIF4G or eIF4G^604–646^-LgBiT level were detected in whole cell lysate (Left, WCL) and m7GTP pulldown (right) from transfected cells in Fig. 1d. (B) Anti-FLAG immunoprecipitation of HEK293 cells transfected only with 4EBP1 constructs followed by western blot analysis with anti-eIF4E and anti-FLAG antibody. (C) 4EBP1 mutants were expressed and purified (Inset: Coomassie stain analysis of purified protein) from *E. coli* and assessed for their ability to bind recombinant eIF4E. Their dissociation constants (K_d_) were determined using a competitive fluorescent anisotropy (FP) assay. (D) Lysates from cells transfected with the 4EBP1 constructs and anti-FLAG immunoprecipitated were additionally probed for 4EBP1 phosphorylation status at threonine 37 and serine 46. eIF4E was also visualised as a loading control. 4EGi1 and 4E1RCat compound titrations on HEK293 cell co-transfected with (E) NanoBit eIF4E:eIF4G^604–646^ PPI system or (F) NanoLuc full length plasmid. (G) Viability of HEK293 cells treated as in (E) and (F) were assessed by measuring intracellular ATP concentrations (CellTiter-GLO, PROMEGA). Luciferase activity was measured as described in “Materials and methods”. The molecular mass of the protein marker is indicated in kDa. All values represent mean ± SD (*n* = 3). (PNG 408 kb)

Additional file 2: **Figure S2.** HEK293 cells transfected with the NanoLuc full length plasmid were treated with titrations of A) PP242, B) Rapamycin and Torin-1, respectively. C) HEK293 cells were treated for 4 h with titrations of PP242, Torin and Rapamycin, respectively, and their viability assessed by measuring intracellular ATP concentrations. Luminescence signals were normalised with those obtained from DMSO vehicle treated cells. All values represent mean ± SD (*n* = 3). (PNG 83 kb)

Additional file 3: **Figure S3.** (A) Western blot analysis showing endogenous level of eIF4E and 4EBP1 in HEK293, PC-3 and PANC-1 cells. Actin was visualised as a loading control. (B) Western blot analysis of eIF4E phosphorylation at serine 209 in HEK293 cells transfected with NanoBit eIF4E:eIF4G^606–646^ system and either treated with CGP57380 or DMSO vehicle control. Both short (SE) and long (LE) exposure of the western blot probed with anti phospho-eIF4ES^209^ are displayed. eIF4E levels were visualised as a loading control. Black arrows indicate SmBit fused eIF4E. (C) PC-3 and (D) PANC-1 cells transfected with the NanoBit eIF4E:eIF4G^606–646^ system were treated with titrations of CGP57380, Torin and PP242, respectively, for 4 h and assessed for effects on their cell viability as assessed by intracellular ATP levels (CellTiter-GLO, PROMEGA). Luminescence signals were normalised with those obtained from DMSO-treated cells. All values represent mean ± SD (*n* = 3). The molecular mass of the protein marker is indicated in kDa. (PNG 164 kb)

Additional file 4: **Figure S4.** (A) Western blot analysis of endogenous levels of eIF4E, eIF4G and 4EBP1 in non-transfected 293FT extracts and associated m7GTP pulldowns of eIF4E containing complexes with differing treatment concentrations of BEZ235. (B) and (C) Compound titrations as described in Fig. 6a were performed on HEK293 cells co-transfected with NanoBit eIF4E:eIF4G^604–646^ system and siRNA Ctrl (B) or siRNA 4EBP1 (C). Compound treatments were performed for 4 h and then cells were assayed for luciferase activity. Data was normalised to DMSO controls and equivalent cell viability experiments. The molecular mass of the protein marker is indicated in kDa. (PNG 177 kb)
