# Supplementary figures and images for: Monitoring flux in signalling pathways through measurements of 4EBP1-mediated eIF4F complex assembly
Source: BMC Biol. 2019 May 22;17:40. doi: 10.1186/s12915-019-0658-0 (PMC6530213; doi:10.1186/s12915-019-0658-0)

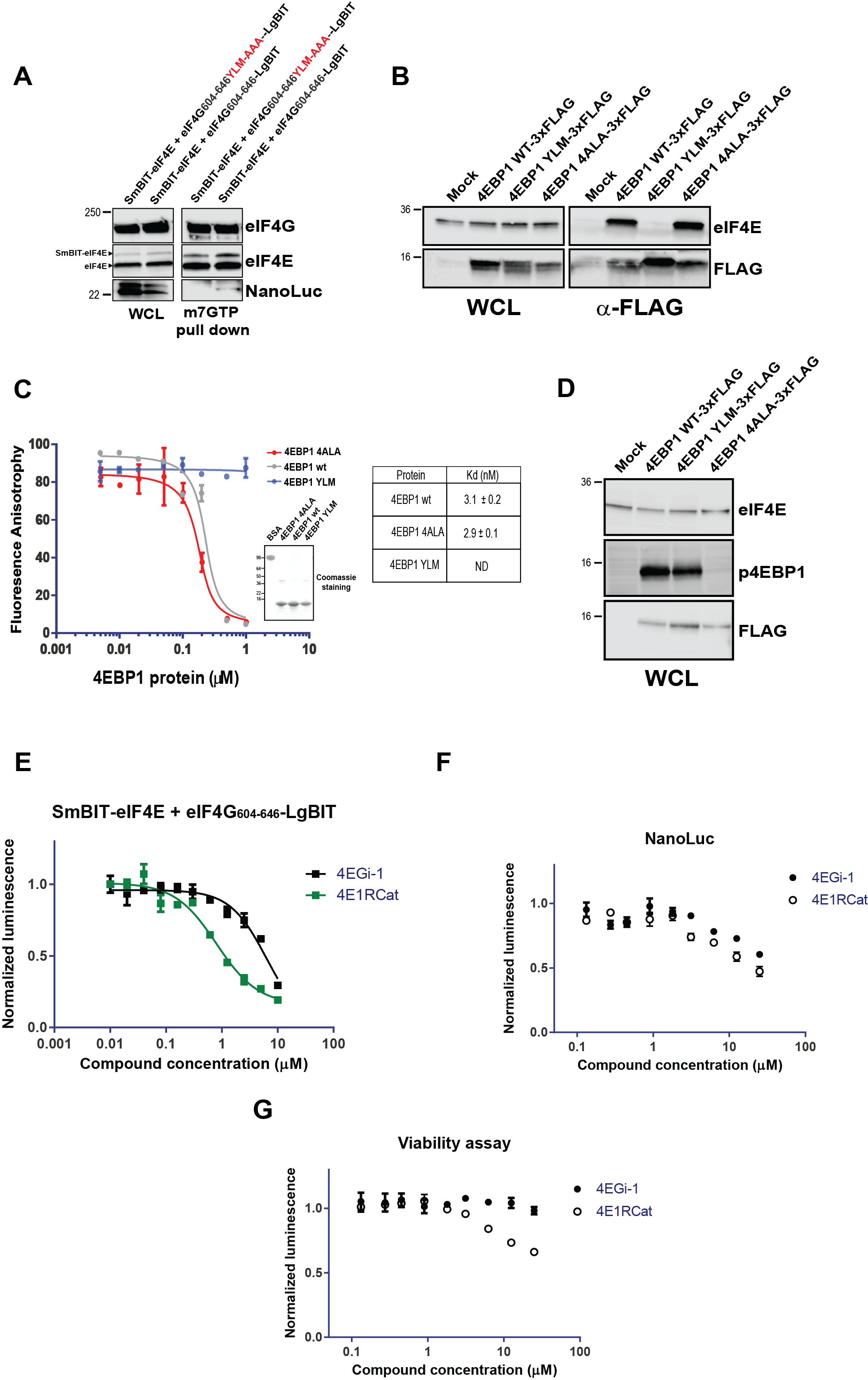

Supplement: Supplementary file 1 — Figure S1. (A) eIF4E and SmBIT-eIF4E (indicated with black arrows), eIF4G or eIF4G604–646-LgBiT level were detected in whole cell lysate (Left, WCL) and m7GTP pulldown (right) from transfected cells in Fig. 1d. (B) Anti-FLAG immunoprecipitation of HEK293 cells transfected only with 4EBP1 constructs followed by western blot analysis with anti-eIF4E and anti-FLAG antibody. (C) 4EBP1 mutants were expressed and purified (Inset: Coomassie stain analysis of purified protein) from E. coli and assessed for their ability to bind recombinant eIF4E. Their dissociation constants (Kd) were determined using a competitive fluorescent anisotropy (FP) assay. (D) Lysates from cells transfected with the 4EBP1 constructs and anti-FLAG immunoprecipitated were additionally probed for 4EBP1 phosphorylation status at threonine 37 and serine 46. eIF4E was also visualised as a loading control. 4EGi1 and 4E1RCat compound titrations on HEK293 cell co-transfected with (E) NanoBit eIF4E:eIF4G604–646 PPI system or (F) NanoLuc full length plasmid. (G) Viability of HEK293 cells treated as in (E) and (F) were assessed by measuring intracellular ATP concentrations (CellTiter-GLO, PROMEGA). Luciferase activity was measured as described in “Materials and methods”. The molecular mass of the protein marker is indicated in kDa. All values represent mean ± SD (n = 3). (PNG 408 kb) [file 12915_2019_658_MOESM1_ESM.png]

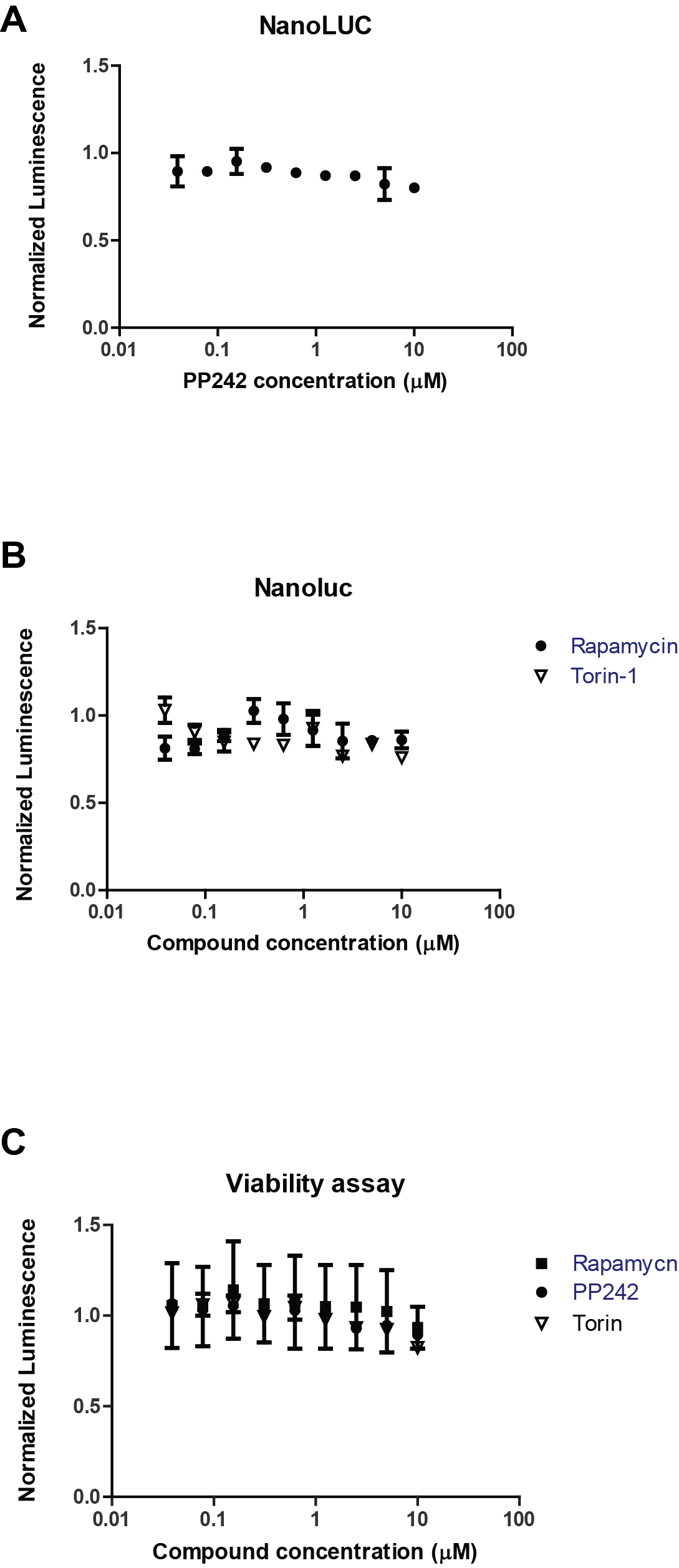

Supplement: Supplementary file 2 — Figure S2. HEK293 cells transfected with the NanoLuc full length plasmid were treated with titrations of A) PP242, B) Rapamycin and Torin-1, respectively. C) HEK293 cells were treated for 4 h with titrations of PP242, Torin and Rapamycin, respectively, and their viability assessed by measuring intracellular ATP concentrations. Luminescence signals were normalised with those obtained from DMSO vehicle treated cells. All values represent mean ± SD (n = 3). (PNG 83 kb) [file 12915_2019_658_MOESM2_ESM.png]

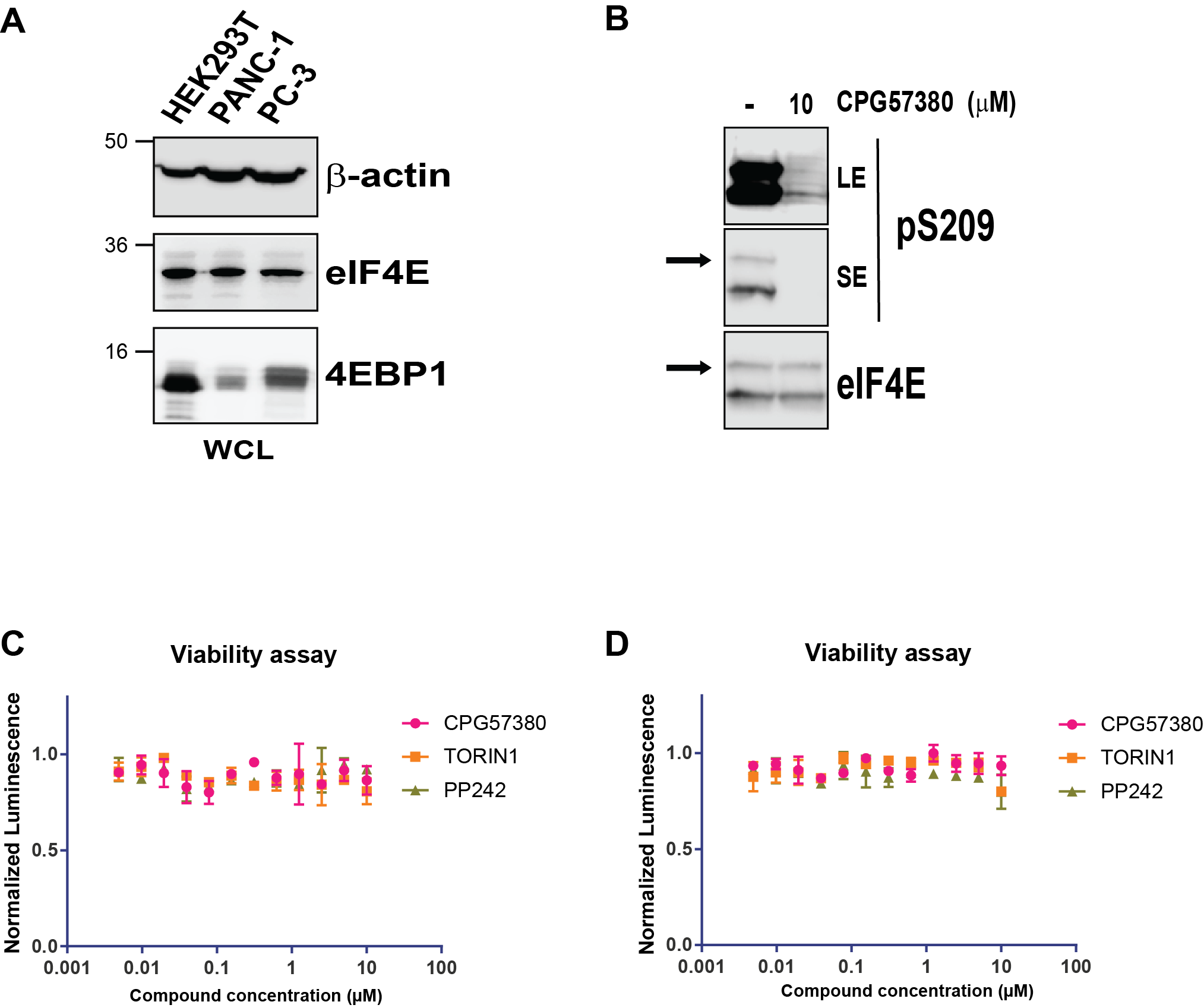

Supplement: Supplementary file 3 — Figure S3. (A) Western blot analysis showing endogenous level of eIF4E and 4EBP1 in HEK293, PC-3 and PANC-1 cells. Actin was visualised as a loading control. (B) Western blot analysis of eIF4E phosphorylation at serine 209 in HEK293 cells transfected with NanoBit eIF4E:eIF4G606–646 system and either treated with CGP57380 or DMSO vehicle control. Both short (SE) and long (LE) exposure of the western blot probed with anti phospho-eIF4ES209 are displayed. eIF4E levels were visualised as a loading control. Black arrows indicate SmBit fused eIF4E. (C) PC-3 and (D) PANC-1 cells transfected with the NanoBit eIF4E:eIF4G606–646 system were treated with titrations of CGP57380, Torin and PP242, respectively, for 4 h and assessed for effects on their cell viability as assessed by intracellular ATP levels (CellTiter-GLO, PROMEGA). Luminescence signals were normalised with those obtained from DMSO-treated cells. All values represent mean ± SD (n = 3). The molecular mass of the protein marker is indicated in kDa. (PNG 164 kb) [file 12915_2019_658_MOESM3_ESM.png]

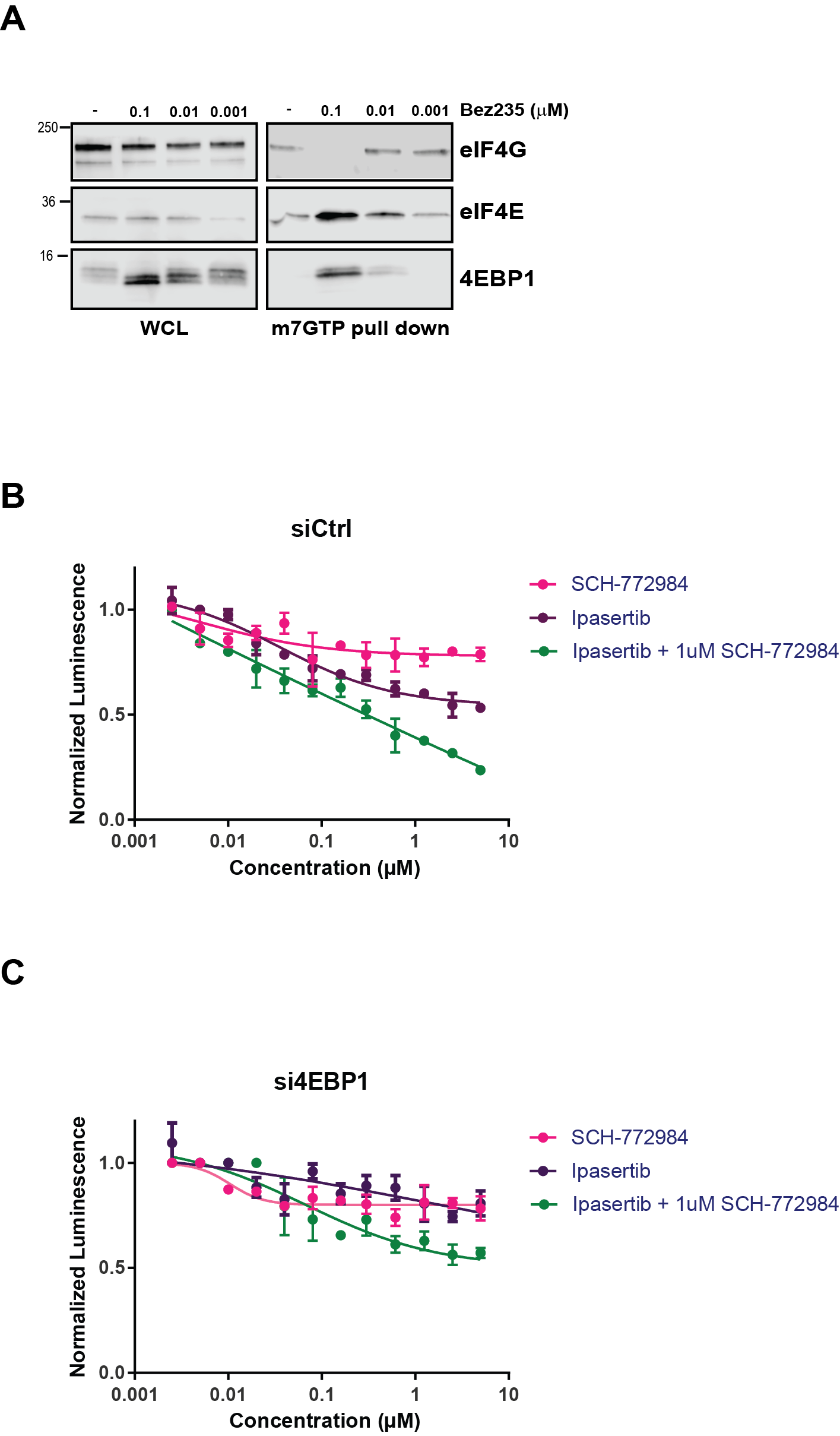

Supplement: Supplementary file 4 — Figure S4. (A) Western blot analysis of endogenous levels of eIF4E, eIF4G and 4EBP1 in non-transfected 293FT extracts and associated m7GTP pulldowns of eIF4E containing complexes with differing treatment concentrations of BEZ235. (B) and (C) Compound titrations as described in Fig. 6a were performed on HEK293 cells co-transfected with NanoBit eIF4E:eIF4G604–646 system and siRNA Ctrl (B) or siRNA 4EBP1 (C). Compound treatments were performed for 4 h and then cells were assayed for luciferase activity. Data was normalised to DMSO controls and equivalent cell viability experiments. The molecular mass of the protein marker is indicated in kDa. (PNG 177 kb) [file 12915_2019_658_MOESM4_ESM.png]
